# MetaCNV - a consensus approach to infer accurate copy numbers from low coverage data

**DOI:** 10.1186/s12920-020-00731-y

**Published:** 2020-06-01

**Authors:** Stefanie Friedrich, Remus Barbulescu, Thomas Helleday, Erik L. L. Sonnhammer

**Affiliations:** 1 Department of Biochemistry and Biophysics, Science for Life Laboratory, Stockholm University, Box 1031, 17121 Solna, Sweden; 2 Department of Oncology-Pathology, Science for Life Laboratory, Karolinska Institutet, Solna, Sweden

**Keywords:** Human genome analysis, Copy number calling, Low coverage data

## Abstract

**Background:**

The majority of copy number callers requires high read coverage data that is often achieved with elevated material input, which increases the heterogeneity of tissue samples. However, to gain insights into smaller areas within a tissue sample, e.g. a cancerous area in a heterogeneous tissue sample, less material is used for sequencing, which results in lower read coverage. Therefore, more focus needs to be put on copy number calling that is sensitive enough for low coverage data.

**Results:**

We present MetaCNV, a copy number caller that infers reliable copy numbers for human genomes with a consensus approach. MetaCNV specializes in low coverage data, but also performs well on normal and high coverage data. MetaCNV integrates the results of multiple copy number callers and infers absolute and unbiased copy numbers for the entire genome. MetaCNV is based on a meta-model that bypasses the weaknesses of current calling models while combining the strengths of existing approaches. Here we apply MetaCNV based on ReadDepth, SVDetect, and CNVnator to real and simulated datasets in order to demonstrate how the approach improves copy number calling.

**Conclusions:**

MetaCNV, available at https://bitbucket.org/sonnhammergroup/metacnv, provides accurate copy number prediction on low coverage data and performs well on high coverage data.

## Background

An important aspect of genome analysis is the study of genetic alterations between individuals in a cohort, or between samples from one individual, for instance to understand cancer progression. One type of genetic alteration is copy number variation (CNV), which describes the fact that a segment of a genome, for example spanning one or more genes, is amplified or deleted.

Up to 10% of the human genome has been estimated to contribute to CNVs, and abnormal copy numbers have been linked to mutation-prone diseases like cancer [1]. Knowledge about CNVs is not only crucial to understanding such diseases, especially with regards to their evolution, but also their effects on various phenotypes.

Next-generation sequencing has revolutionized the possibilities to study inter- and intra-individual genome alterations. For accurate CNV analysis, typically tissue samples with 200–500 ng of DNA are required for sequencing with high coverage [2]. However, new generations of sequencing techniques arise and new approaches to sequence even single cell genomes are being developed that only require 50 ng of DNA [2–4]. Copy number callers applicable to sequenced samples with very small amounts of DNA would create the possibility to investigate CNVs of smaller areas within a larger tissue sample, such as regions microdissected by laser, and thus offer spatial information of CNVs within formerly bulk-sequenced samples.

CNVs can be detected experimentally with various assays, such as comparative genomic hybridization (CGH, e.g. bacterial artificial chromosomes (BAC) array), fluorescent in situ hybridization (FISH), and genotyping array. Another option is calling CNVs from genome sequences, although calling correct copy numbers this way is a challenging task. To develop a solution, four different approaches have emerged, each built on certain assumptions and each with advantages and disadvantages [5, 6].

Callers purely based on a read coverage approach (i) predict copy number changes using the read coverage of a segment of the genome relative to the coverage of the whole genome. To achieve good results for both deletions and amplifications, this approach requires high sequencing depth (Fig. S1). Further, short CNVs are often missed. The majority of existing copy number callers apply this concept, for example ReadDepth [7], CNV-seq [8], cn. MOPS [9], Control-FREEC [10], and CNVnator [11].

Callers based on a paired-end (read-pair) mapping approach (ii) predict copy numbers based on changes in the insert size of paired-end reads. This approach requires paired-end sequenced data and only considers those pairs of reads where both ends of a pair have been mapped which decreases the number of reads and thus the amount of data that can be used for copy number calling. It is appropriate to identify structural variants in general (e.g. inversions, inter and intra-chromosomal translocations) [5, 12]. Discordantly mapped reads (i.e mapping span or paired-end orientation are inconsistent with the reference genome) indicate a structural variant [13]. The paired-end mapping approach is relatively independent from the sequencing depth. A popular package is BreakDancer [14].

The split-reads approach (iii) takes only discordant mapped reads; that is, only one mate was aligned concordantly. For the unmapped read, alignment to a reference genome is reattempted by splitting the read and aligning both parts separately. A popular package doing this is PINDEL [15].

Callers that apply combinations of the above described approaches, i.e. hybrids, represent the fourth type (iv). Combining a paired-end read and a read depth approach seems to be the most beneficial [5], for instance used by the callers SVDetect [16] and CNVer [17].

The rapidly growing field of single cell DNA (scDNA) sequencing challenges calling of variants, e.g. copy numbers, for individual cells having coverages ~1x. Examples of such callers specified for scDNA are Ginkgo [18] and SCNV [19]. They are both based on a read depth approach and correct for GC content bias. A limitation with these methods is that in order to deal with technical artefacts introduced by single cell sequencing leading to high noise, they require pools of at least 3 cells for calibration or normalisation. SCNV is further an example of adapting an established bulk method, SeqCBS, to scDNA data [19]. Another method is Lumpy [20], a structural variant caller applicable to low coverage data. However, it only outputs cnv types, i.e. deletion or amplification.

However, in general callers require relatively high sequence coverage to achieve good results, or if specialised in scDNA, callers require multiple cells to be applied on. Although higher sequencing depth can be achieved with technologies like polymerase chain reaction (PCR), this leads to unevenly distributed copies of unique molecules, and fewer unique molecules with increasing sequencing depth influencing mutation calling. We here introduce MetaCNV, a method that combines different approaches in order to create a caller that is sensitive enough to detect CNVs in low coverage data (below 10x), even one single cell, but also works on normal and higher coverage data (above 30x and 100x, respectively). It is a generally applicable method that in contrast to previous low coverage methods can be applied to single samples.

## Algorithm and implementation

### Implementation

MetaCNV v1.4 is intended for use on unix operating systems. The graphical user interface was created with the GTK+ toolkit, a free library available for the majority of current unix distributions (Additional file 1).

### MetaCNV algorithm

MetaCNV combines the prediction of copy number callers based on different approaches and builds a consensus to achieve higher prediction accurateness. Some copy number callers (e.g. SVDetect) require a matched sample, for example to be able to distinguish somatic (only detected in the primary sample) from germline mutations (also found in the matched sample, e.g. blood). One of the current input callers for MetaCNV, SVDetect, was run with three different matched sample versions: a matched blood sample, a simulated normal sample with 20x read coverage, and a simulated null (simNull) alignment with constant zero read coverage, to test if this choice affects the prediction accurateness and increases sensitivity especially on low coverage data.

**Fig. 1:**
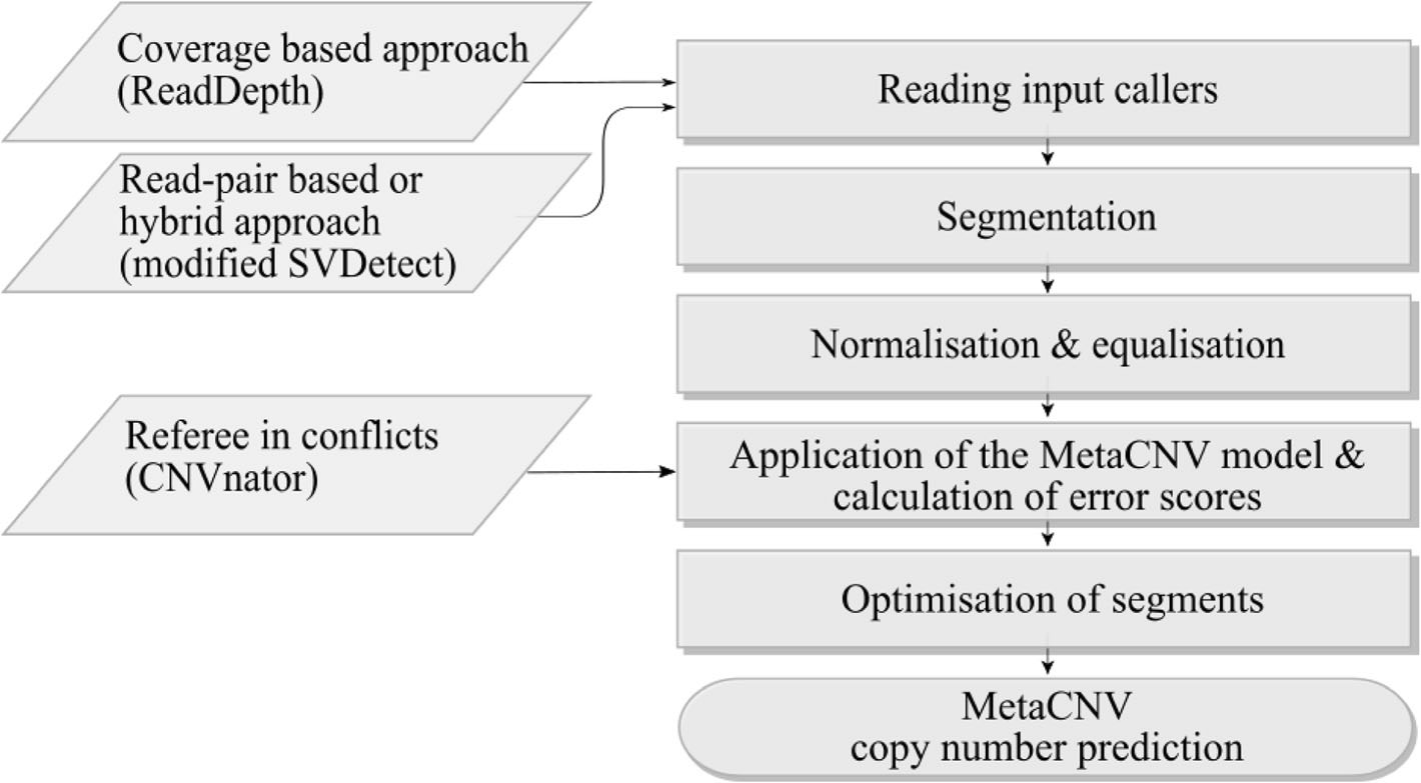
MetaCNV workflow to call copy numbers. MetaCNV requires as input an SVDetect prediction using a simulated null alignment (simNull) as matched sample (Fig. S19)

Running MetaCNV comprises five steps (Fig. 1).

#### Choice of current input callers

The current input callers for MetaCNV were chosen due to their relatively good prediction accurateness on low coverage data (Figs. 4 & S13). Further, they belong to different calling approaches which perform differently depending on the type of a CNV. In low coverage data, we observed that ReadDepth v0.9.8, which applies a read depth approach, predicts mid-sized (10^4^–10^6^ bp) and larger deleted segments (> 10^6^ bp) more accurately than other callers, whereas amplifications were more accurately predicted by SVDetect v1.3, a representative of the hybrid approach.

Finally, a caller’s prediction coverage (Fig. S7A, Table S3) is considered. Only ReadDepth and CopyCat [21] predict gapfree CNVs for an entire genome. Gapfree means that for each base pair of an investigated genome, a copy number is calculated which resulted in a high prediction coverage. SVDetect predicted for more than 90% of the genomes. The prediction coverage of Control-FREEC and CNVnator varied in cancer cell data sets from 33 to 65% and 35 to 80%, respectively.

ReadDepth is built on the coverage-based approach, predicting copy numbers gapfree for the whole genome. It applies a negative binomial distribution to approximate an overdispersed Poisson distribution [7]. Further, it outputs absolute copy numbers for equally-sized, non-overlapping bins (Table S15). The optimal bin size is calculated by ReadDepth, but can be indirectly adapted by changing the false discovery rate.

SVDetect is a tool used to detect general structural variants and is based on the hybrid approach for copy number calling, meaning both the coverage and any change in the insert size of read-pairs are considered when inferring copy numbers [16]. SVDetect copy numbers are not predicted gapfree, and in contrast to ReadDepth and CNVnator it requires for copy number calling a matched normal sample with which to relate the studied sample (Table S16).

CNVnator v0.3.2, like ReadDepth, is based on the coverage approach, but with an additional mean-shift approach that produces a probability distribution function from the coverage data, and links each data point, i.e. bin, to its maxima. Further, CNVnator is calibrated using the extensive validation done by the 1000 Genomes Project [11, 22]. Similar to SVDetect, it does not calculate copy numbers for the entire genome. As with SVDetect, the bin size can be modified by the user. Despite achieving good prediction accurateness on high coverage data, CNVnator predicts extremely high copy numbers on low coverage data (Figs. S8, Table S17). However, it still produced reliable classification of segments into deletions and amplifications for low coverage data as accurately as SVDetect and ReadDepth, and complements their predictions (Fig. S13, Table S17). Based on this, ReadDepth, SVDetect, in combination with CNVnator as a referee for conflicts between ReadDepth and SVDetect were chosen as input callers for MetaCNV.

#### Matched sample in SVDetect

When SVDetect was run as part of MetaCNV, a simulated null sample with zero read coverage (simNull) was used. The simNull alignment as matched sample is a novel idea to increase sensitivity and to remove an additional source of noise for low coverage data. If comparing a low coverage sample with a matched sample having high coverage it will disturb a correct copy number calling. Ideally, a matched sample should have the same constant coverage as the sample to investigate. With current tools this constant coverage on each base pair of the genome is not achievable (Fig. S21). Therefore, we developed the novel idea of a simulated null alignment containing zero coverage, and thus no noise.

When SVDetect was run outside of MetaCNV for comparison, two different types of such matched samples were tested: a matched normal sample (matchedNormal) and a simulated normal sample with constant read coverage (simNormal).

The matched normal was taken from the cancer cell lines HCC1187 and HCC2218 (Table 1) for which such samples are available (blood samples HCC1187BL and HCC2218BL). The simulated normal was inferred using Pirs [24].

**Table 1:** Cancer cell lines used for accurateness testing. Coverage and number of deleted and amplified genes extracted from COSMIC are presented. Copy numbers were called from sequenced genomes and experimentally confirmed with PICNIC [23]

Cancer cell line	Coverage	# of amplified genes	# of deleted genes	Total # of genes with CNVs	Ratio amplified/deleted genes
HCC1187	104x	154	46	200	3.48
HCC2218	93x	200	36	236	5.55
MCF7	62x	49	30	79	1.63
PC3	76x	179	222	401	0.81
Single-cell SKBR3	6 stepwise merged single cells with 1x to 6x	161	29	190	5.55

#### Segmentation of the genome

**Fig. 2:**
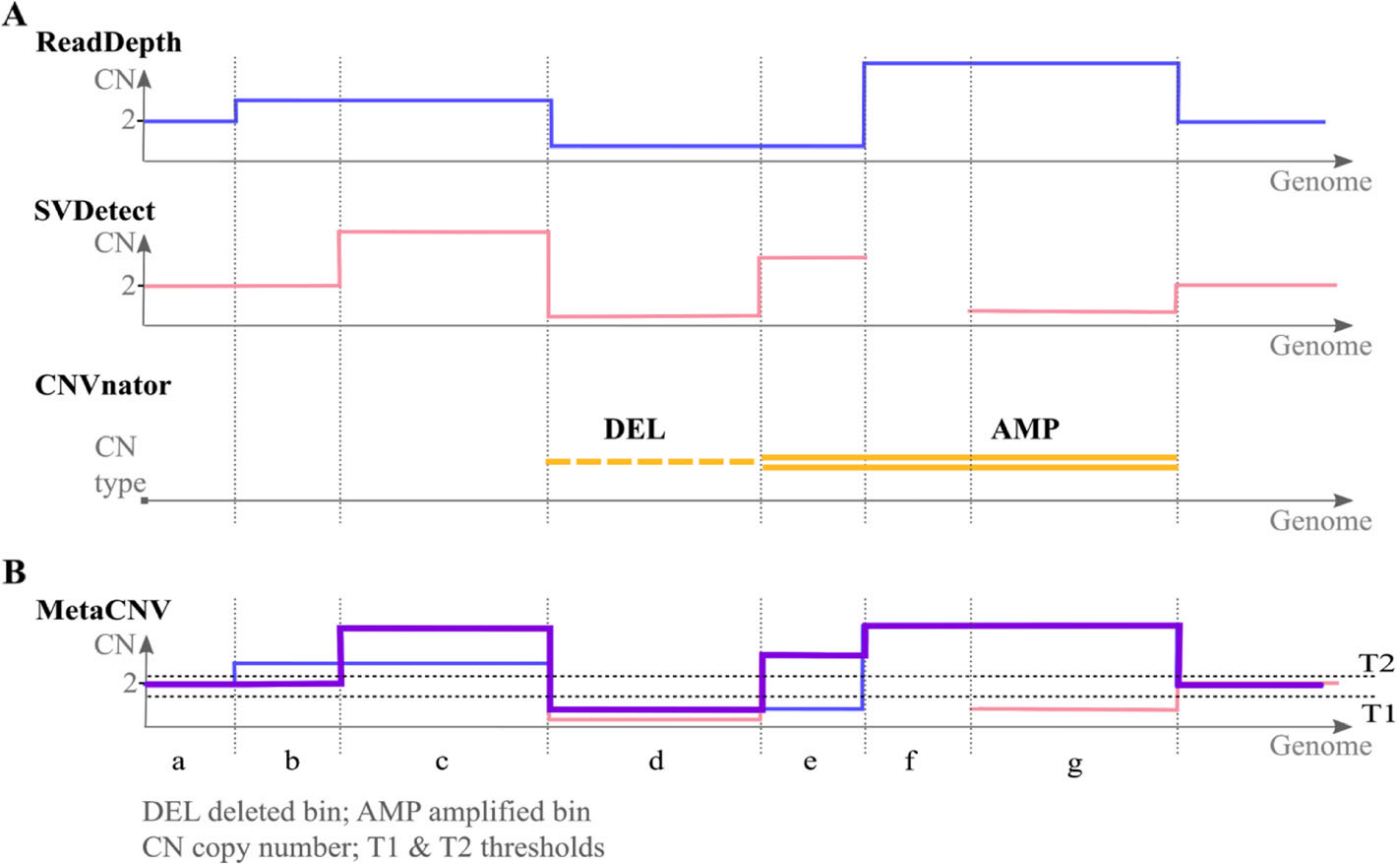
Genome segmentation by MetaCNV. **a** Segmentation of the genome according to the bins and breakpoints from the input callers. **b** Consensus segment prediction by MetaCNV is marked as a thick line. Bins c is predicted as amplified and bin d as deleted. Here there is no conflict between the input callers, hence the consensus is that c is amplified and d is deleted. Bin f is not predicted by SVDetect, but because ReadDepth predicts it as an amplification, this becomes the consensus. Bins b, e and g have conflicting ReadDepth and SVDetect predictions. CNVnator judges that e and g are amplifications, hence this becomes the consensus. Bin b is set to CN 2 because only ReadDepth predicts it as an amplification and CNVnator makes no prediction (Table S18)

ReadDepth divides a given genome into bins of a calculated minimum or a multiple of the minimum bin size. The bin size can only be manipulated indirectly by increasing the false discovery rate. For both, the high and low coverage study of sequenced single cells, it was set to 0.01 (default). SVDetect accepts a bin size given by the user. Several bin sizes were tested for SVDetect but the best results were achieved with a bin size of 400 bp and no bin overlap. Based on the calculated bin sizes from both callers hidden in the start and end position in the output files, new bins were calculated with different sizes while also considering all breakpoints given by the input callers (Fig. 2). At the end of the MetaCNV prediction process, bins with a similar copy number to one decimal place were merged into one segment.

#### MetaCNV model

**Fig. 3:**
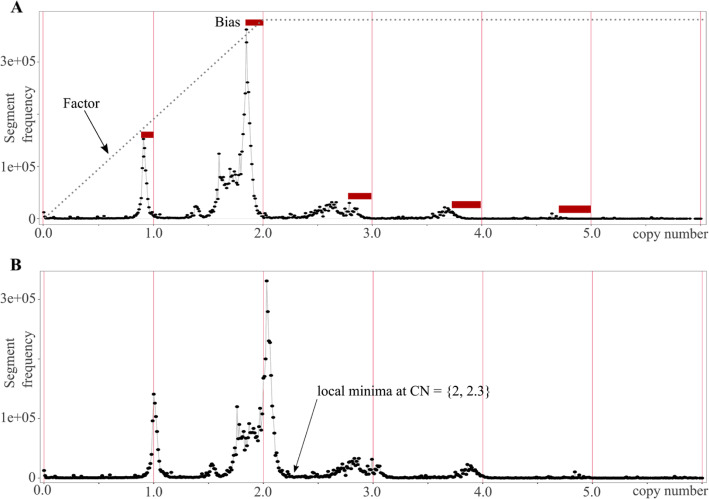
Distribution of absolute copy numbers. Copy numbers were called with ReadDepth of cancer cell line HCC2218 after segmentation (6,867,679 segments with an average segment length of 450 bp). **a** The bias occurs around each integer copy number and is lower for *CN*_*RD*_ ~ 1, but increases for *CN*_*RD*_ ~ 2. The factor for normalisation is a linear function (eq. 1b) which serves to adjust the bias for different *CN*_*RD*_. **b** Distribution of absolute copy numbers called with ReadDepth after normalisation

The MetaCNV model contains rules to be applied to each bin, depending on the predicted copy numbers of the input callers representing different approaches of copy number calling; the strength of rules is in the decreased risk of overfitting. In general, MetaCNV accepts deletions and normal copy numbers if they are predicted by a coverage approach (current ReadDepth), and amplifications if they are predicted by a read-pair approach or hybrid approach of coverage and read-pair (current SVDetect). More detailed rule descriptions are listed in Table S18. To distinguish between deletions and normal copy numbers from amplifications, two thresholds *T1* and *T2* are introduced. *T2* is the local minimum of the frequency of copy numbers produced by ReadDepth between 2 and 2.3; the other threshold *T1* is calculated as 2 * *P* - *T2* where *P* is the ploidy value (Fig. 3).

Due to gaps in the predictions from at least one current input caller (no copy number predicted) or conflicting predicted copy numbers from both callers, the MetaCNV model comprises additional rules and includes CNVnator as the referee in such conflicting cases.

#### Normalisation of input data

When developing a consensus approach, the results of the considered input callers need to be normalised in order to compare their results correctly. The detected bias in the predicted copy numbers of each input caller was corrected with a copy-number-dependent normalisation. The bias (systematic error) is the difference between frequency peak and ploidy (Fig. 3, eqs. 1), where CN is the predicted copy number by a caller, *CN*_*norm*_ is the normalised CN, and *P* is the ploidy value which is for the autosomes 2 and for the allosomes either 2 if female or 1 if male. The maximum absolute bias correction is 0.5.

1 $$ {CN}_{norm}= CN+\left( factor\times bias\right) $$

1a $$ bias=P- CN\; at\;\mathit{\max}\ \left( frequency\;(CN)\right) $$

1b $$ factor=\mathit{\min}\;\left(\frac{1}{2}\times CN;1\right) $$

#### Converting log_2_ values into absolute copy numbers

In general, callers predict copy numbers either as absolute copy numbers or as log ratio values which can be converted into absolute copy numbers with.

2 $$ {CN}_{corrected}=P\times {2}^{CN_{\mathit{\log}2, corrected}} $$

Absolute copy numbers reflect the number of repeats of the sequence [7]. The log_2_ values represent log_2_ transformed ratios to a matched sample or the ploidy *P* [6].

When MetaCNV is run with input from SVDetect using a simulated normal sample, the formula above (eq. 2) is applied to convert log_2_ values into absolute copy numbers for each bin. Before this, the log_2_ values *CN*_*log2*_ are corrected with a genome wide median:

2a $$ {CN}_{\mathit{\log}2, corrected}={CN}_{\mathit{\log}2}- median\ \left({CN}_{\mathit{\log}2}\right). $$

When MetaCNV is run with input from SVDetect using a simulated null alignment, the predicted values are accepted as copy numbers. This produces *CN*_*log2*_ values that are always non-negative, because in the case of the simulated null alignment, they are relative to 0. Further, a distortion was observed which increases exponentially with the true amplification. This distortion is corrected with an equalizer factor *q* (Fig. S20, eq. 3). The net effect corresponds to a back log transformation that is calibrated to result in absolute copy numbers.

3 $$ {CN}_{meta}={CN}_{\mathit{\log}2}\times q $$

3a $$ q={\left(1+\frac{1}{100}\times {CN}_{\log 2}\right)}^{0.75\kern0.62em {CN}_{\log 2}} $$

#### Calculating error scores

Each MetaCNV bin is annotated with an error score e mirroring the prediction similarity of the input callers. The score depends on the consensus of the input callers per bin and is averaged for the optimised segments considering the bin length per segment. The error score per bin is calculated as the squared absolute copy number difference between ReadDepth and SVDetect:

4 $$ {e}_{bin}={\left({CN}_{RD}-{CN}_{SVDetect}\right)}^2 $$

For bins where ReadDepth did not predict a copy number, we assume CN = 0. ReadDepth predicts copy numbers gapless for a genome. Although, rare large gaps happen to occur but they are caused by non-sequenced regions which not contain genetic regions according to the reference genome GRCh38 [25]. If SVDetect did not predict a copy number, CN = *Ploidy* is used. SVDetect does not predict copy numbers gapless for a genome although the coverage is sufficient in case of the high coverage data and no other obvious reason could be identified. (Pre-processing and application of calling methods, Suppl., Tables S19 and S20).

### Accurateness evaluation

To evaluate the accurateness of a caller, its result needs to be compared to true copy number variations. Different ways were taken to set such a gold standard: simulated data, for example for the validation of CNV-seq; clinical data with experimentally confirmed CNV, e.g. for the validation of CNVnator; or cancer cell lines, e.g. for the validation of Control-FREEC and ReadDepth.

Further, the comparison between true and predicted copy number has either been performed for segments, e.g. validation of Control-FREEC and ReadDepth, or for genes [26]. Different accurateness measures have been applied, including accuracy, false discovery rate, F1-score [27], receiver operating characteristic with true positive and false positive rates [28], Spearman correlation, and root mean squared error [29].

Finally, absolute copy numbers, log_2_ ratio values or the class of a copy number [27], which is either a deletion or an amplification, were considered. Absolute copy numbers are decimal or integer values mirroring the number of repeats of the sequence [7] whereas the log_2_ ratios stand for log transformed ratios to a matched sample or the ploidy [6].

The prediction accurateness of a regression model, which is the case if comparing copy numbers, can be evaluated using the mean squared error (MSE, eq. 5) and mean absolute error (MAE). In general, an error based measure calculates the difference between a true and a predicted value, which in this case is the difference between true and predicted copy number per gene. The mean absolute error presents the average error, whereas the mean squared error combines systematic and random error into one value [30]. It also penalizes outliers: each distance is squared, and larger distances thus get more weight.

The true copy number per gene, *x*_*i*_, was compared with the predicted one, ${\hat{x}}_i$, by calculating the residuals ${x}_i-{\hat{x}}_i$ for all genes *N*.

5 $$ \mathrm{Mean}\kern0.17em \mathrm{squared}\kern0.17em \mathrm{error}\  MSE=\frac{1}{N}\sum \limits_{i=1}^N{\left({x}_i-{\hat{x}}_i\right)}^2 $$

To avoid outlier penalty we compare additionally the prediction accurateness with the mean log ratio error:

6 $$ \mathrm{Mean}\ \log\ \mathrm{ratio}\ \mathrm{error}\  MLRE=\frac{1}{N}\sum \limits_{i=1}^N{LR}_i,{LR}_i= abs\left( In\left(\frac{x_i+1}{{\hat{x}}_i+1}\right)\right) $$

If a caller predicts highly different copy numbers for genes of a genome having the same true copy number but also predicts similar copy numbers for genes with different true copy numbers, then the analysis of copy number variations can become difficult. Therefore, copy number callers were also evaluated by the variance of the residuals, which mirrors how close the predicted values surround each unique true copy number (eq. 7. For each unique true copy number (integer) value, the variance of the predicted values was calculated, however, default elements were replaced with the residuals *z*_*i*_ (the difference between actual *x*_*i*_ and predicted value ${\hat{x}}_i$). The variances are then averaged for the total number of unique true copy numbers M.

7 $$ \mathrm{Variance}\ \mathrm{of}\ \mathrm{residuals}\ {\sigma}_{Res}^2=\frac{1}{M}\sum \limits_{j=1}^M\left(\frac{1}{N}\sum \limits_{i=1}^N{\left({z}_i-\overline{z}\right)}^2\right) $$

Matthew’s correlation coefficient (MCC) [31] can be applied for classification models and is especially applicable for unbalanced ratios of the four confusion matrix categories, which is the case for the cancer cell lines used in this paper (ratio of amplified/deleted genes from 0.8 to 5, Table 1). To define the classes of deletions and amplifications for ploidy *P* = 2, copy numbers > 2.75 were set as amplifications and copy numbers < 1.75 were set as deletions, while values in between were set as normal. In order to deal with these three classes (deletion, normal, amplification), the values for true positives, true negatives, false positives and false negatives were micro-averaged. This means that for example the true positive value *TP* is equal to *TP*_*class1*_ + *TP*_*class2*_ + *TP*_*class3*_ .

The prediction accurateness of several copy number callers and MetaCNV was evaluated using known amplified or deleted genes that were publicly available along with well-studied cancer cell lines (Additional file 2). MetaCNV was developed for low coverage data. Therefore, MetaCNV’s accurateness was verified on single sequenced cells of a cancer cell line (SKBR3) having 1x to 6x read coverage. Additionally, the accurateness was assessed on four cancer cell lines with normal and high coverage (62x to 104x coverage, Table 1). The range of copy numbers among the cancer cell lines was limited. To validate MetaCNV on a wider range of copy numbers and also genome-wide, mutated genomes of different coverages were simulated. MetaCNV’s accurateness was compared with other callers’ accurateness by MLRE and MCC (MSE, MAE, and Spearman’s correlation in Figs. S11, S13, S14).

For each caller, we map the output (segments and corresponding copy numbers) per cancer cell line and simulated genome to the human assembly GRCh38 Ensembl (release 84) [25]. Due to different segment sizes, this mapping resulted in one or several predicted copy numbers per gene. In such cases, the total copy number per gene was the sum of the weighted copy numbers, depending on their segment length within the gene (Fig. S9). There were gaps in the prediction of SVDetect, CNVnator, and Control-FREEC, that is, no copy number for a segment was called. Such gaps within a gene were filled using the ploidy value. Some segments of the cancer cell lines given by COSMIC [32] (Cosmic, Suppl.) did not cover a gene completely. In these rare cases, the gene was reduced to the covered segment length within this gene.

### Cancer cell lines

Cancer cell lines, like the HeLa cancer cell line, which can theoretically be divided and replicated indefinitely, contain cells taken from e.g. naturally-occurring cancer tissues [33]. They are publicly available and well-studied objects; mutations like CNVs and other structural variations are experimentally confirmed [34].

The use of cancer cell lines is advantageous due to the fact that the reviewed callers have to perform on real sequencing data, and the result can be compared to known CNVs. Using cancer cell lines can also be a disadvantage because of the lower heterogeneity found in them; high heterogeneity is a common characteristic of cancerous clinical samples. Further, variance and grades of deletions and amplifications are lowered, since a cancer cell line contains only a limited number of cells, compared to a bulk-sequenced clinical sample containing million of cells. Despite the disadvantages, testing a copy number caller’s accurateness on cell lines is easy, transparent, and replicable.

The cancer cell lines HCC1187 (with matched blood sample), HCC2218 (with matched blood sample), MCF7, PC3, and SKBR3 (Tables 1, S1, S2, Figs. S2-S4, S7B) were chosen to compare MetaCNV’s accurateness with other callers’ accurateness. The sequenced DNA of a single cell of SKBR3 (1x per cell) by using a novel method to sequence both, genome and transcriptome of the same single cell [4], was used as low coverage data. The sequenced and aligned single cell genomes were stepwise merged to present increasing coverages.

### Simulated mutated genomes

For each of four coverages (1x, 2x, 5x, and 10x), three mutated human genomes were simulated (gw1, gw2, lcd). The paired-end reads for the chromosomes 1–22 were generated using CNVsim v0.9.2, aligned using Bowtie2 v2.2.9 [35], and converted, sorted and indexed using Samtools v1.2 [36]. For each of the three genomes, 30 to 50 segments per chromosome and each segment with a copy number unequal to 2 and a length of 10 kbp to 100 kbp were generated (Figs. S5 and S6). The simulated genomes were mapped to GRCh38 (Table 2). Two simulated genomes (gw1, gw2) were used to compare the callers’ results for a genome wide copy number prediction. CNVnator and Control-FREEC only gave partial predictions, see Table S4. The third simulated genome (lcd) was therefore used to compare the callers’ results on a reduced data set comprising segments for which a prediction of all callers (MetaCNV, SVDetect, CNVnator, ReadDepth, Control-FREEC) was available. This, however, limited the range of copy numbers from 0 to 4.

**Table 2:** Simulated mutated genomes used for accurateness testing. The simulated data sets gw1 and gw2 were used to compare the prediction results for a genome-wide prediction. The simulated data sets lcd was used to compare the prediction results for genes for which a prediction of all tested callers was available

Simulation	Depth of coverage	# of amplified genes	# of deleted genes	Total # of genes	Ratio amplified/ deleted genes
sim 1x gw1	1.1x	283	559	13,906	0.51
sim 1x gw2	1.1x	236	573	13,920	0.41
sim 1x lcd	1.5x	25	30	330	0.83
sim 2x gw1	2.2x	247	560	13,937	0.44
sim 2x gw2	2.2x	254	588	13,922	0.43
sim 2x lcd	2.9x	42	44	16	0.95
sim 5x gw1	5.7x	230	580	13,987	0.40
sim 5x gw2	5.7x	251	593	13,949	0.42
sim 5x lcd	7.3x	62	48	14	1.29
sim 10x gw1	11.3x	263	560	13,882	0.47
sim 10x gw2	11.3x	240	551	13,865	0.44
sim 10x lcd	14.9x	54	49	7	1.10

## Results

The consensus copy number caller presented here, MetaCNV, combines a number of primary callers by rules that optimally harness the strengths of each method. MetaCNV’s and other callers’ accurateness were evaluated by calling copy numbers for cancer cell lines and comparing the results with experimentally confirmed deleted or amplified genes in the COSMIC database. To consider a higher number of mutated genes and a wider range of copy numbers, MetaCNV’s and the other callers’ accurateness was also evaluated on three simulated mutated human genomes for each of the coverages 1x, 2x, 5x, and 10x.

True and predicted absolute copy numbers were compared by the mean log ratio error (MLRE) across all genes. We also calculated Matthew’s correlation coefficient, MCC, on the predicted classes of deleted and amplified genes. This measure evaluates how well callers can differentiate between amplifications and deletions in general.

### Prediction accurateness on low coverage data

**Fig. 4:**
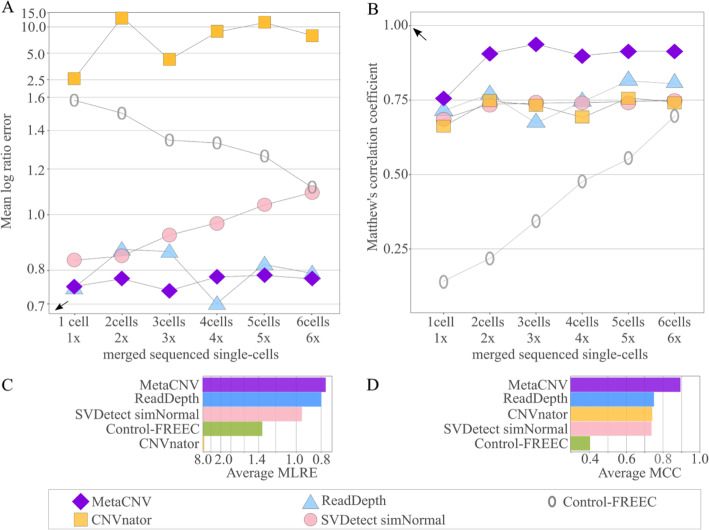
Prediction accurateness of MetaCNV on low coverage data, compared to CNVnator, Control-FREEC, ReadDepth, and SVDetect. **a** and **b** present the benchmark results with MLRE and MCC per merged alignment. **c** and **d** show the overall results averaged across all benchmarked alignments

We assessed copy number prediction accurateness on a single-cell sequenced cancer cell line (SKBR3) with stepwise merged alignments of one additional cell, and stepwise increased coverage from 1x to 6x. MetaCNV was compared to the popular copy number callers CNVnator, Control-FREEC, ReadDepth, and SVDetect. In all benchmarks, MetaCNV was the most accurate method. MetaCNV outperformed the other methods in the majority of coverages in the MLRE benchmark. In all MCC benchmarks, MetaCNV was the most accurate method. MetaCNV was the most robust method as it was the top performer for MLRE, MSE, MAE, and MCC in the majority of the coverage levels (Figs. 4, S13- S15, Tables S8-S10, Additional files 7, 8, 9, 10, 11 and 12).

CNVnator produced only a reliable classification of segments into deletions and amplifications; absolute copy numbers were not usable. For example for the SKBR3 cell line, sequenced single cell nr. 1, copy numbers for regions of deletions ranged from 0 to 2664 and copy numbers for regions of amplifications ranged from 0 to 44,084 (Fig. S8). Increasing coverage from 1x to 6x improved the accurateness only for Control-FREEC, a coverage based approach, although it never reached the accurateness of its competitors.

### Prediction accurateness on high coverage data

MetaCNV’s accurateness on cancer cell lines with high read coverage was compared to the accurateness of other callers which are CNVnator, CopyCat, Control-FREEC, ReadDepth, and SVDetect.

**Fig. 5:**
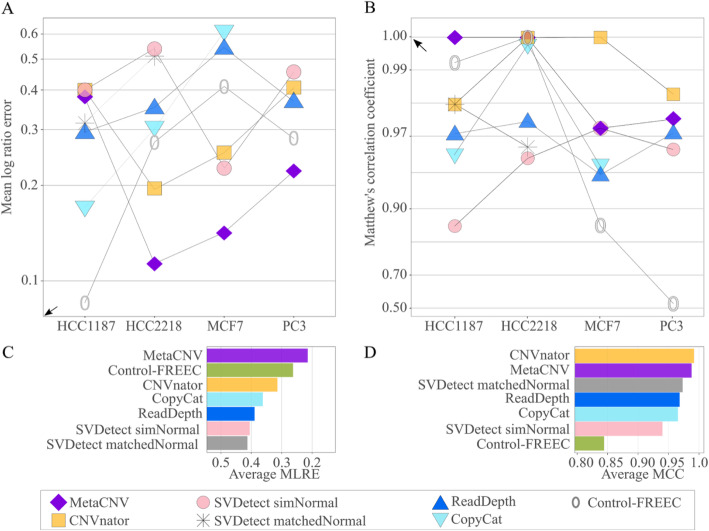
Prediction accurateness of MetaCNV and other copy number callers for high coverage data. **a** and **b** present the average benchmark results per cancer cell line. SVDetect using matchedNormal could only be performed for HCC1187 and HCC2218, for which matched blood samples were available. **c** and **d** show the overall results averaged across all benchmarked cell lines

Although MetaCNV was designed for low coverage data, it also performed well for high coverage data. In three of four tested cancer cell lines, it was the best performer (Figs. 5, S12, Tables S11-S14, Additional files 3, 4, 5 and 6). Several predictors reached an MCC near 1.0 in two cell lines, hence there was no clear winner there (Figs. 5, S10). To present an overall accurateness, MLRE and MCC were averaged over the four tested cancer cell lines per caller (Fig. 5c & d). MetaCNV showed the best overall accurateness with MLRE and second best with MCC, only 0,004 behind CNVnator.

### Prediction accurateness on simulated mutated genomes

MetaCNV’s predictive accurateness on simulated mutated human genomes was compared to the predictive accurateness of CNVnator, SVDetect, ReadDepth, and Control-FREEC. The simulated datasets comprise the predictions genome-wide and reduced to genes for which an output from all callers was available. Each dataset was simulated in different coverages (1x, 2x, 5x, 10x). MetaCNV performed best in all tested simulated datasets using MLRE and MCC (Figs. S16- S18, Tables S5-S7); in two datasets (2x-gw2 and 5x-lcd) MetaCNV and ReadDepth performed equally well using MLRE.

### The novel simNull alignment as matched sample

SVDetect, one of the current input callers for MetaCNV, requires a matched sample for copy number calling. For the cancer cell lines HCC1187 and HCC2218, a matched normal blood sample was used (HCC1187BL, HCC2218BL). Additionally, a normal sample was simulated with constant coverage. However, a simulated normal sample also contains noise and variance in coverage which negatively influences copy number calling for low coverage data. Further, comparing a low coverage sample (e.g. 3x) with a matched sample having higher coverage (e.g. 30x) will lead to an insensitive result. Therefore, we developed the novel idea of a simulated null alignment containing zero coverage, and thus no noise, to improve the sensitivity of copy number calling.

SVDetect using a simulated null alignment creates one of the inputs for MetaCNV. For comparison, MetaCNV was also run with input from SVDetect using a simulated normal sample. MetaCNV with a simulated null alignment achieved better overall results than with the simulated normal alignment when evaluated using MSE and MLRE (Fig. S19). This was true for both low and high coverage data, although the effect was much stronger for low coverage data.

However, just replacing the matched simulated normal sample with a simulated null alignment in SVDetect did not always improve the prediction for high coverage data, see Fig. S19.

Taken together, the superior accurateness of MetaCNV stems from: (i) combining the prediction of multiple callers to form a consensus, (ii) considering that the reliability of a copy number depends on the approach the caller was based on, and (iii) for low coverage data, using a matched sample with zero instead of normal coverage.

## Discussion

To investigate smaller areas within a tissue section or even sequenced single cells, callers are needed that are able to detect CNVs in low coverage alignments. We present MetaCNV a copy number caller specialised in low coverage data. MetaCNV is based on a consensus approach combining different calling approaches and achieved a better prediction accurateness on low coverage data than other reviewed callers. MetaCNV also performed well on high coverage data. (Figs. 4 & 5).

In low coverage data, we observed that a read depth approach is better in predicting deleted segments, whereas amplifications were more accurately predicted by representatives of the paired-end mapping or hybrid approach. However, current callers, including those that employ a hybrid model, apply the developed calling model to each type of variation (deletions and amplifications). MetaCNV considers several calling approaches and builds a consensus based on rules to avoid overfitting, which results in predicting CN more accurately. Due to the different approaches of the current input callers (ReadDepth, SVDetect, and CNVnator), MetaCNV is biased towards mid-sized and large deletions and short and mid-sized amplifications.

To identify somatic structural variants, callers such as SVDetect require a matched normal sample. In order to increase sensitivity for low coverage data, we developed the novel idea of a simulated null alignment used as matched sample. MetaCNV requires the calling result from SVDetect using this simNull as matched sample. For demonstration, MetaCNV was also tested with the input from SVDetect using a simulated normal, however, with a simNull performing a better prediction. The effect of a simulated null alignment compared with a simulated normal or matched normal is low if applied on high coverage data. In contrast, the impact increased immensely for low coverage data where, on the one hand, additional noise disturbs the prediction accurateness, and on the other hand, high sensitivity is required to predict reliable copy numbers. The simNull alignment leads to a distortion of predicted copy numbers that increases with the copy number. This distortion is corrected in a simple way, but with more alignments having different read coverages, a more sophisticated approach could lead to a further improvement in prediction.

The evaluation in this study was done using an error benchmark comparing true and predicted value per instance (gene), the mean log ratio error (MLRE), and Matthew’s correlation coefficient (MCC) to assess how well a caller can distinguish between deletions and amplifications. The benchmarks mirror that coverage-based approaches are highly dependent on read coverage, and show that MetaCNV outperforms other callers on low coverage data and performs well on high coverage data.

It was not possible to include specialized low coverage scDNA callers in the benchmark. Ginkgo and SCNV can not be run for single samples and Lumpy v0.2.13 does not output quantitative CNV values. CNV-seq v0.2–8 gave ambiguous copy numbers for overlapping bins. We could however run Ginkgo using all SKBR3 single cell samples, which gives it considerably more information than the callers presented in the benchmark have. Despite this, Ginkgo achieved worse accurateness than MetaCNV for all six SKBR3 cells when evaluated with MCC, and worse or equal accurateness for four cells when evaluated with MLRE.

## Conclusions

We hypothesized that a CNV caller that is based on a consensus approach and considers the mutation type in the prediction, can predict copy numbers more reliably and is sensitive enough to be applicable for low coverage data. MetaCNV, the presented CNV caller, predicted CNs more accurately on low coverage data than other reviewed callers. MetaCNV also performed well on high coverage data.

Using MetaCNV it is possible to investigate CNVs of smaller areas within a larger tissue sample, such as regions microdissected by laser or sequenced single cells, for which only very small amounts of DNA are available.

## Availability and requirements

Project name: MetaCNV

Project home page: https://bitbucket.org/sonnhammergroup/metacnv

Operating system(s): unix

Programming language: C++

Other requirements: GTK + -2.0 or higher

Licence: GNU LGPL

Any restrictions to use by non-academics: none

## Supplementary information

**Additional file 1.** MetaCNV Readme Manual to run MetaCNV.

**Additional file 2.** CCLs knownCNVs Supplementary table containing genes per cancer cell line, incl. Copy number, segment length and evaluation length.

**Additional file 3.** CNVcalling HCC1187 CNV calling results for HCC1187.

**Additional file 4.** CNVcalling HCC2218 CNV calling results for HCC2218.

**Additional file 5.** CNVcalling MCF7 CNV calling results for MCF7.

**Additional file 6.** CNVcalling PC3 CNV calling results for PC3.

**Additional file 7.** CNVcalling SKBR3–1 cell CNV calling results for SKBR3–1 cell.

**Additional file 8.** CNVcalling SKBR3–2 cells CNV calling results for SKBR3–2 cells.

**Additional file 9.** CNVcalling SKBR3–3 cells CNV calling results for SKBR3–3 cells.

**Additional file 10.** CNVcalling SKBR3–4 cells CNV calling results for SKBR3–4 cells.

**Additional file 11.** CNVcalling SKBR3–5 cells CNV calling results for SKBR3–5 cells.

**Additional file 12.** CNVcalling SKBR3–6 cells CNV calling results for SKBR3–6 cells.

**Additional file 13.** [23, 24, 32, 35–38].

## Data Availability

The cancer cell lines analysed in the current study together with European Nucleotide Archive (ENA) accession numbers and direct links are listed in Table S1. The tumour normal WGS data sets provided by Illumina (HCC1187 & HCC2218) are available at https://basespace.illumina.com/datacentral. The human assembly GRCh38 Ensembl (release 84) files were downloaded from ftp://ftp.ensembl.org/pub/release-84/.

## Abbreviations

CNV: Copy number variation
MCC: Matthew’s correlation coefficient
MSE: Mean squared error
MAE: Mean absolute error
MLRE: Mean log ratio error
