# Supplementary material for: MetaCNV - a consensus approach to infer accurate copy numbers from low coverage data
Source: BMC Med Genomics. 2020 Jun 1;13:76. doi: 10.1186/s12920-020-00731-y (PMC7268502; doi:10.1186/s12920-020-00731-y)
Supplement: Supplementary file 13 — Additional file 13. [23, 24, 32, 35–38]. [file 12920_2020_731_MOESM13_ESM.docx]

# Supplementary


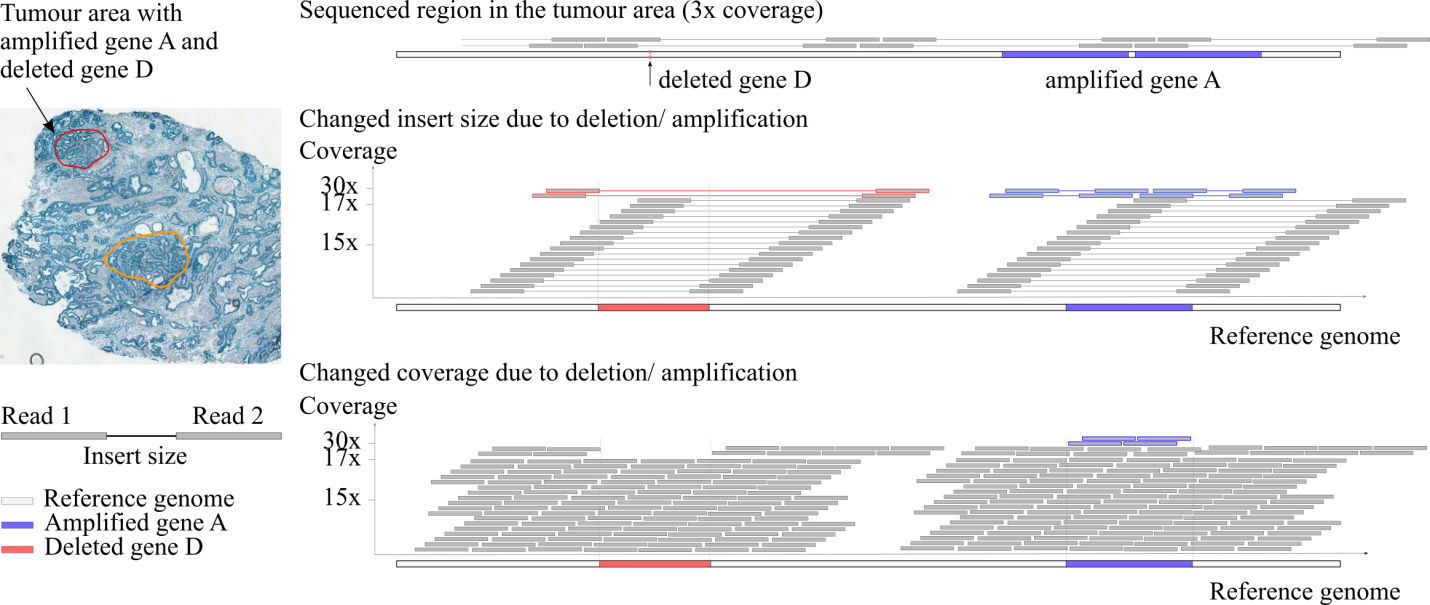


**Figure S1. Copy number calling using a read depth and a read-pair approach.** As an example, a tissue sample harbouring a cancerous area having 10% of the tissue size was bulk sequenced with 30x coverage [35]. The cancerous area is, thus, on average covered by 3x. Using a read-pair approach, only correctly aligned read-pairs were considered. In case of a deleted gene *D*, the majority of read pairs were neglected as only one read mate covered the deleted gene. If the deleted segment is smaller than the insert size of the read pairs, the deletion can be detected. In contrast, amplification are easier to detect with a read-pair approach due to more read pairs that can be considered. Concerning the read depth approach, a deletion leads to an obvious change of coverage. The total length of the mutated sequence reduces to 1/CN in the aligned genome. A coverage approach works better for segment lengths that exceed the genome-wide noise of coverage variation.

## Cosmic

COSMIC is “the catalogue of somatic mutations in cancer” [31] which collects and offers the mutation profiles of cancer cell lines, along with other important information. Especially of interest here is the genome-wide copy number analysis, which was obtained with the Affymetrix SNP6.0 array and analysed with the PICNIC algorithm. [34]⁠

PICNIC is an algorithm specially developed for cancer genomes with an aneuploid nature. It uses the SNP allele signal from Affymetrix genome-wide SNP6.0 array data and predicts “major and minor copy numbers, complex cancer genotypes, homozygous deletion, amplification, and regions of LOH [loss of heterogeneity] with good break-point accuracy” [34]. To complete the task, the algorithm first distinguishes copy number data and genotype intensities, then applies a Bayesian hidden Markov model (HMM) to identify segments with a similar integer allelic copy number.

COSMIC applies a filter to only display high-level copy number variations which results in a non-exhaustive range of genes with amplifications or deletions [31].

For each of the five cancer cell lines known amplifications and deletions were downloaded from COSMIC. The list comprises the affected gene, the copy number segment with its stop and start positions, and the segment’s copy number (integer value).

In case a segment did not completely cover a gene length, actual and predicted copy numbers were only compared for this reduced length which is covered by the segment.

**Table S1.** ENA Cancer cell lines accession numbers and links.

| Cancer cell line | Links to Illumina and the European Nucleotide Archive (ENA) study accession number: sample accession number | Number of lanes |
| --- | --- | --- |
| HCC1187 | <https://basespace.illumina.com/sample/46814013/HCC1187C> | unknown; downloaded from Illumina already in aligned format (*.bam) |
| HCC1187BL | <https://basespace.illumina.com/sample/46814012/HCC1187BL> | unknown; downloaded from Illumina already in aligned format (*.bam) |
| HCC2218 | <https://basespace.illumina.com/sample/46814011/HCC2218C> | unknown; downloaded from Illumina already in aligned format (*.bam) |
| HCC2218BL | <https://basespace.illumina.com/sample/46814010/HCC2218BL> | unknown; downloaded from Illumina already in aligned format (*.bam) |
| MCF7 | [PRJNA317928](https://www.ebi.ac.uk/ena/data/view/PRJNA317928): [SAMN04631739](https://www.ebi.ac.uk/ena/data/view/SAMN04631739) | 1 lane |
| PC3 | [PRJNA361315](https://www.ebi.ac.uk/ena/data/view/PRJNA361315): [SAMN06231154](https://www.ebi.ac.uk/ena/data/view/SAMN06231154) | 4 lanes |
| SKBR3 single cell | [PRJNA266282](https://www.ebi.ac.uk/ena/data/view/PRJNA266282): [SAMN03160687](https://www.ebi.ac.uk/ena/data/view/SAMN03160687), [SAMN03160689](https://www.ebi.ac.uk/ena/data/view/SAMN03160689), SAMN03160702, SAMN03160698, SAMN03160694  SAMN03160701 | 1 lane per cell |


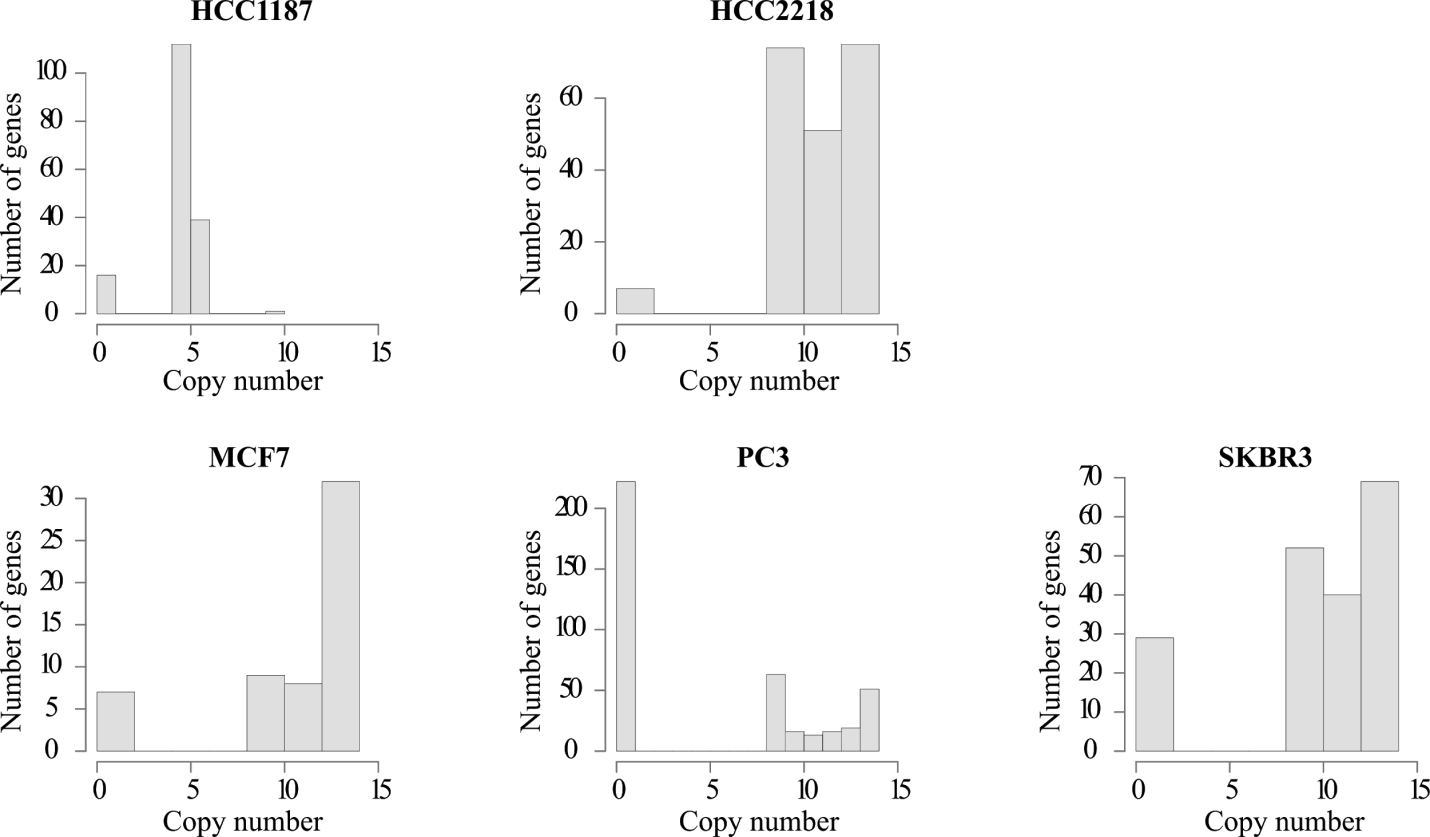


**Figure S2. The distribution of copy numbers per cancer cell line given by COSMIC**


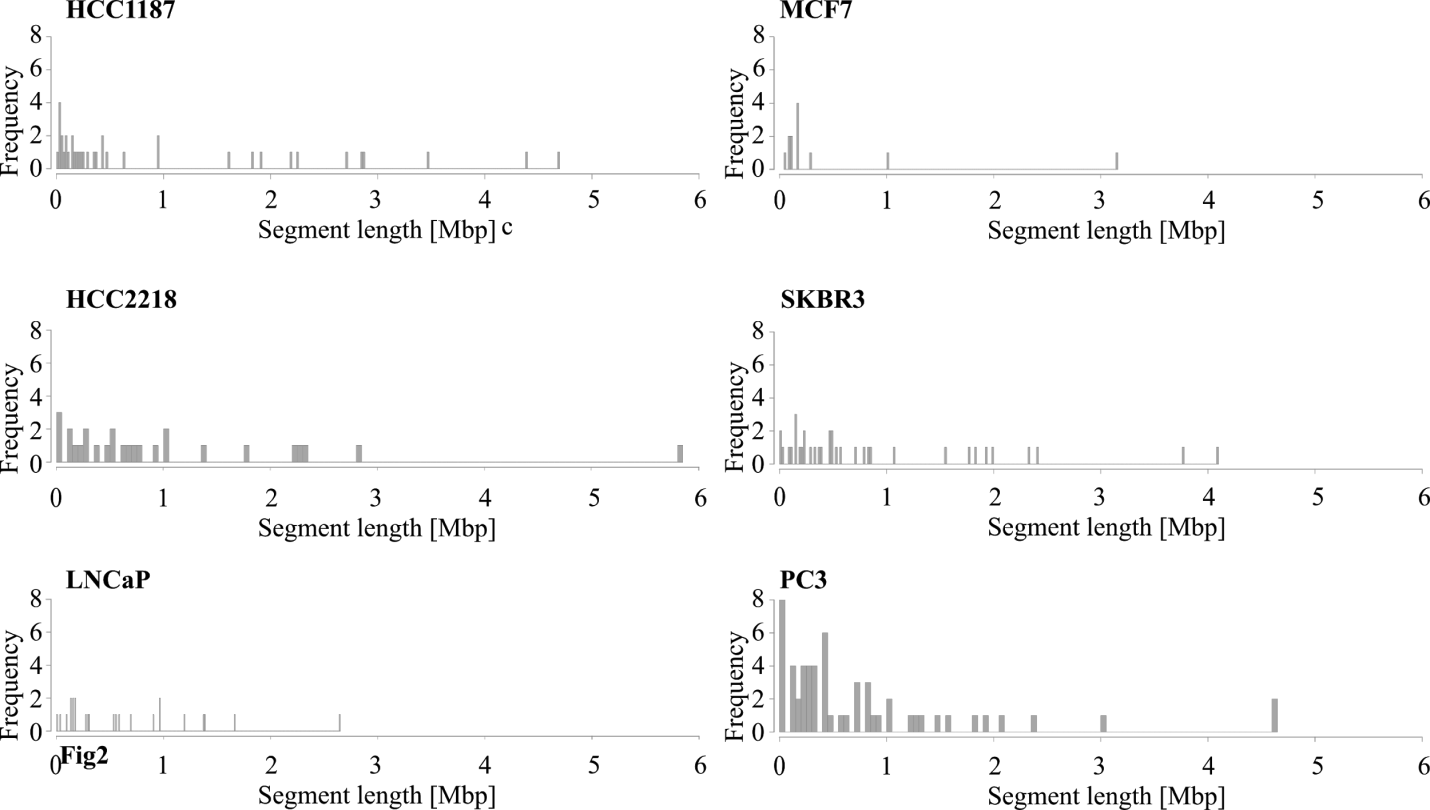


**Figure S3. The distribution of segment lengths per cancer cell line given by COSMIC**

**Table S2.** Numbers of fastq reads and mappability for cancer cell lines MCF7, PC3 and SKBR3. HCC1187 and HCC2218 were downloaded from Illumina in an aligned format.

| Cancer cell line | Number of fastq paired reads | Mappability with Bowtie2 |
| --- | --- | --- |
| MCF7 | 1,217 Mio | > 99 % |
| PC3 | 800 Mio | > 99 % |
| SKBR3 cell 3 | 27.6 Mio | 84 % |
| SKBR3 cell 10 | 30.1 Mio | 55 % |
| SKBR3 cell 13 | 33.7 Mio | 72 % |
| SKBR3 cell 20 | 34.5 Mio | 30 % |
| SKBR3 cell 25 | 45.2 Mio | 44 % |
| SKBR3 cell 26 | 47.7 Mio | 41 % |


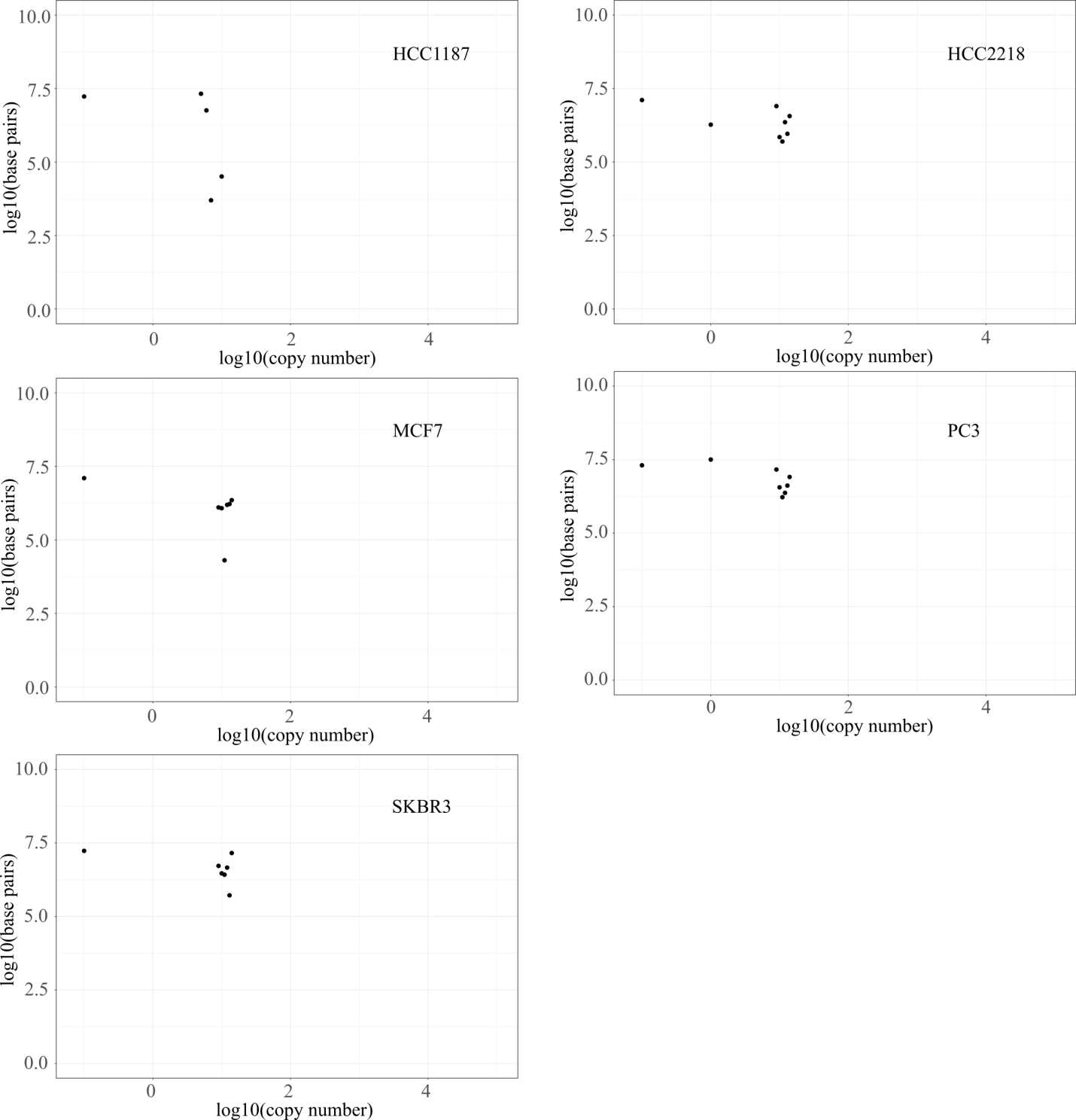


**Figure S4. Distributions of copy numbers given by COSMIC.** The total number of base pairs per copy number is shown; considered were all segments given by COSMIC. Copy numbers < 0.1 were replaced by 0.1 to limit the log(*CN*) value to -1.


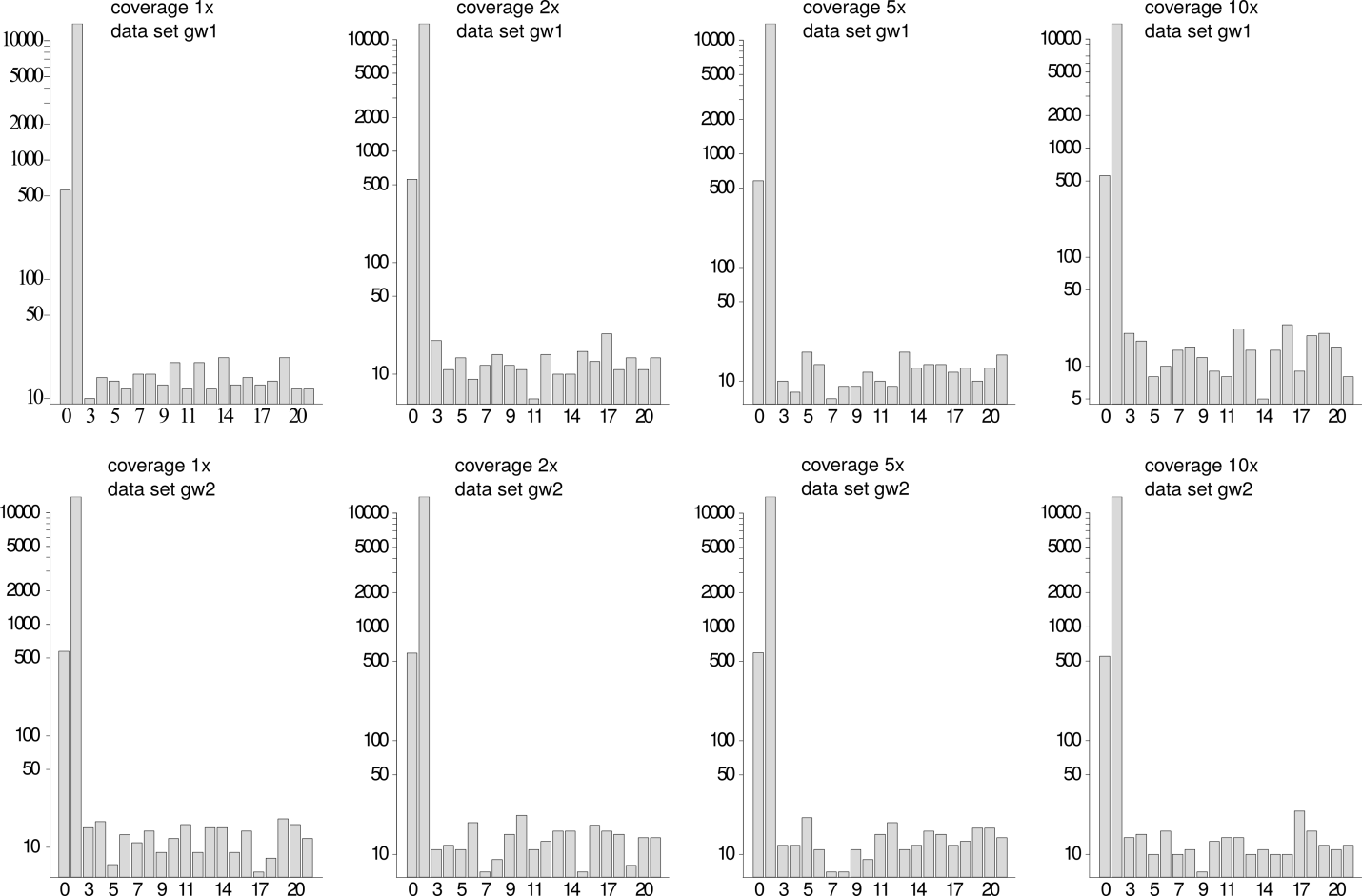


**Figure S5.** Distributions of copy numbers for the simulated mutated genomes (genome wide data sets 1 on the top row and data sets 2 on the bottom row).


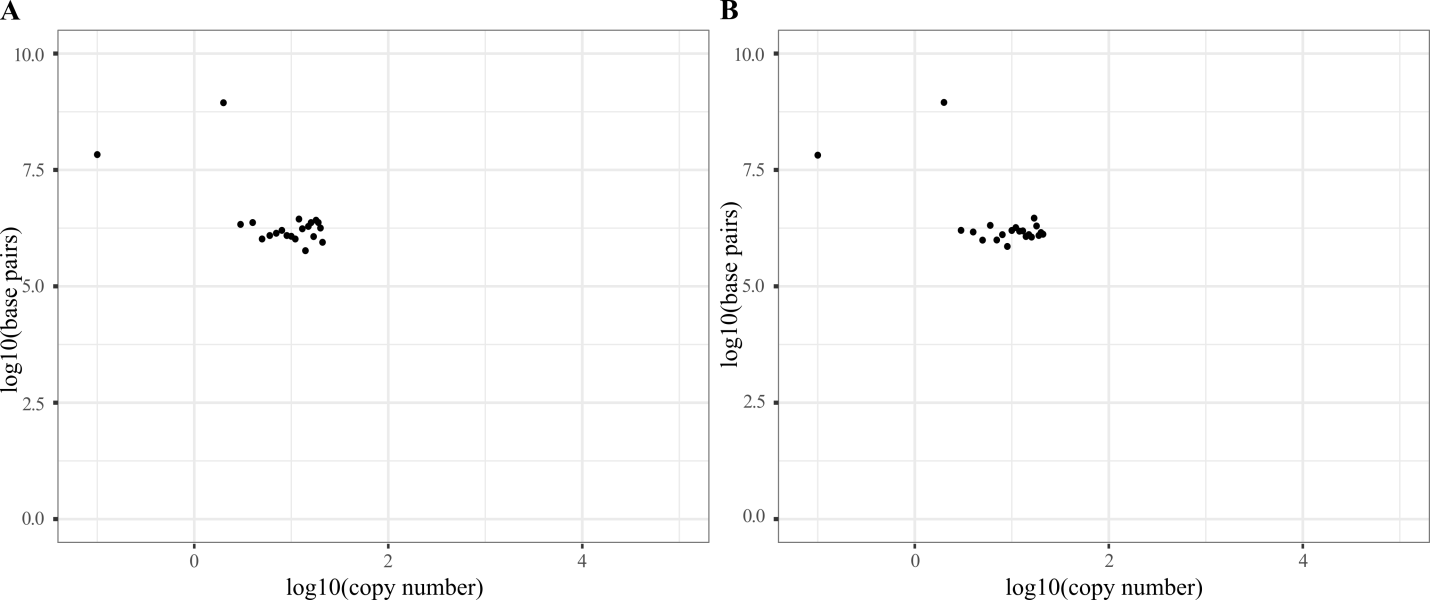


**Figure S6.** Distributions of copy numbers for the simulated mutated genomes used for genome-wide prediction of copy numbers. The distribution is shown for the two datasets gw1 (**A**) and gw2 (**B**) of the simulated coverage 1x. The datasets of the remaining coverages 2x, 5x, and 10x are similar. Copy numbers < 0.1 were replaced by 0.1 to limit the log(*CN*) value to -1.

## Callers’ prediction coverage

**Table S3.** Caller coverage per cancer cell line (genome length 3,088,386,401 bp). Each of the investigated callers introduced gaps in the tested genomes. For larger gaps, a visual check of the alignment revealed non-sequenced regions (no read coverage) which did not contain genetic regions either.

| Caller | HCC1187 | HCC2218 | MCF7 | PC3 | SKBR3 single cell |
| --- | --- | --- | --- | --- | --- |
|  | [%] | [%] | [%] | [%] | [%] |
| ReadDepth | 96.5 | 96.5 | 96.5 | 99.5 | 97.1 |
| SVDetect | 93.1 | 93.1 | 94.1 | 94.5 | 93.7 |
| CNVnator | 80.3 | 35.5 | 39.6 | 57.0 | 40.9 |
| MetaCNV | 99.9 | 99.9 | 99.9 | 99.9 | 99.9 |
| Copycat | 98.6 | 98.6 | 98.6 | NA | NA |
| Control-FREEC | 49.5 | 33.3 | 40.3 | 65.7 | 47.1 |


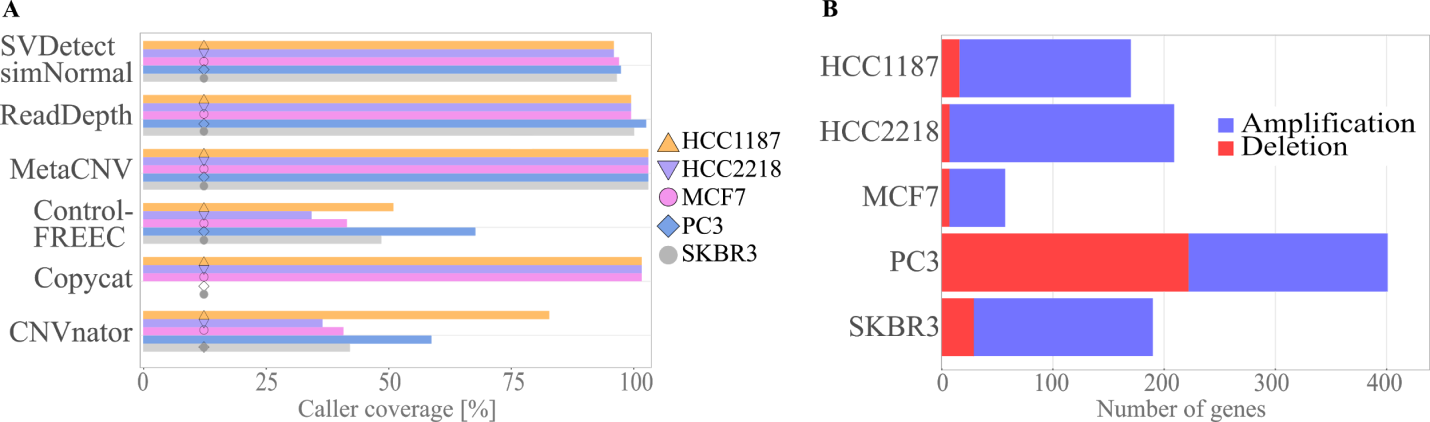


**Figure S7**. **Prediction coverage and distribution of deletions and amplifications per cancer cell line A**. The prediction coverage per caller and cancer cell line is presented. The evaluated callers predicted copy numbers for the genomes gapfree or with gaps. Using ReadDepth, MetaCNV, or CopyCat more than 95% of the genomes were covered with copy number predictions **B.** For each cancer cell line, the number of true amplified and deleted genes are shown which are the basis to calculate MLRE, MSE, MAE, MCC, and Spearman’s correlation.


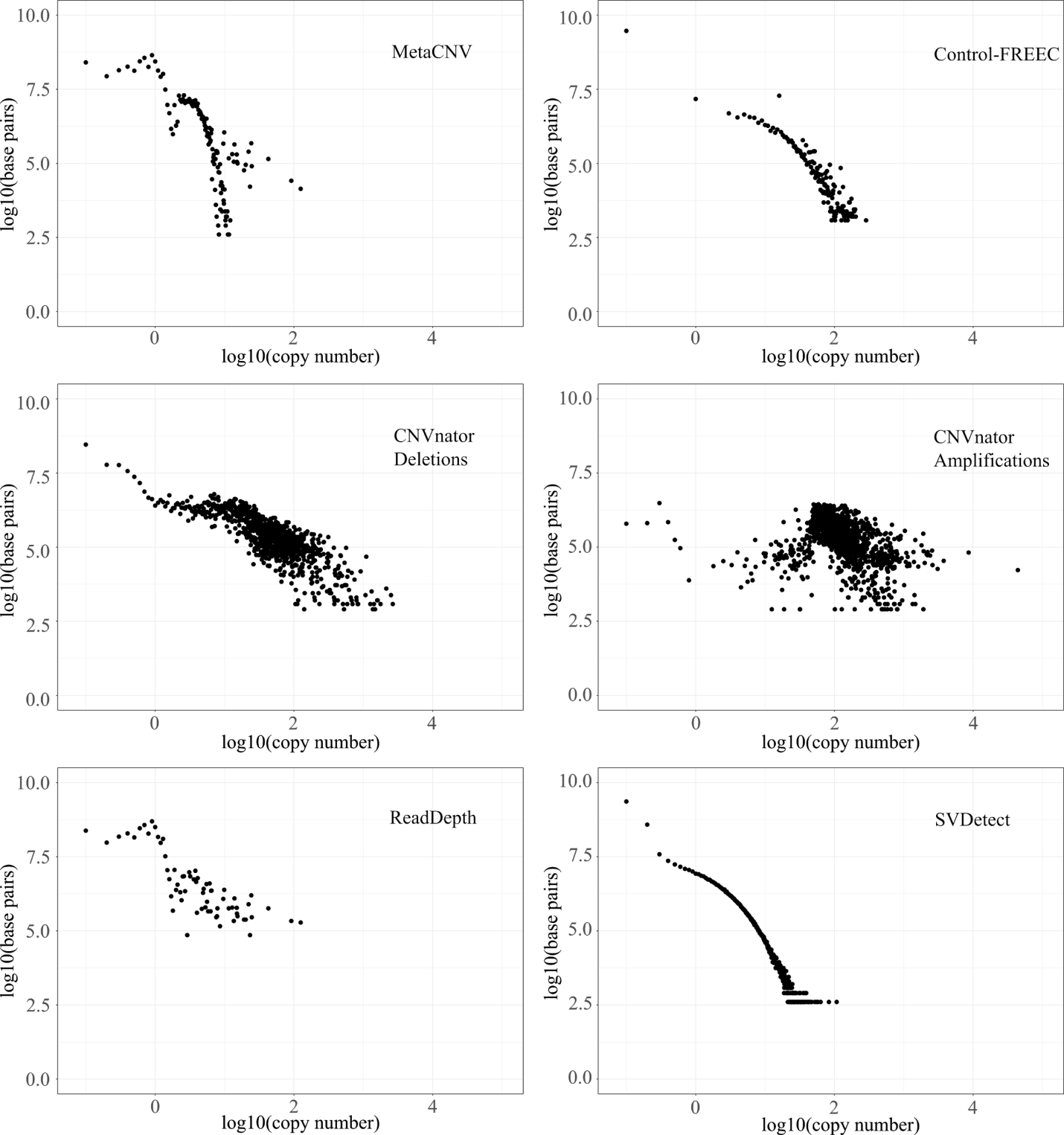


**Figure S8. Distributions of copy number predictions for low coverage data.** Each method was run on single cell 1 in the cell line SKBR3. Per copy number rounded to one decimal, the total number of base pairs per copy number is shown. Copy numbers < 0.1 were replaced by 0.1 to limit the log(*CN*) value to -1. CNVnator predicts both, a copy number and the CNV type; the distribution of predicted copy numbers is shown per predicted CNV type.

**Table S4.** Prediction coverages per caller and simulated mutated genomes for genome-wide prediction. MetaCNV, SVDetect, and ReadDepth predicted copy numbers for the whole genomes, whereas Control-FREEC only covered ~10% of the genomes. CNVnator predicted only for the simulated 1x samples genome-wide.

| Caller | Depth of coverage | Prediction coverage  Data set gw1 | Prediction coverage  Data set gw2 | Prediction coverage  Data set lcd |
| --- | --- | --- | --- | --- |
| CNVnator | 1 | 99 | 99 | 99 |
| CNVnator | 2 | 15 | 15 | 16 |
| CNVnator | 5 | 15 | 15 | 15 |
| CNVnator | 10 | 15 | 15 | 16 |
| Control-FREEC | 1 | 10 | 10 | 10 |
| Control-FREEC | 2 | 12 | 11 | 10 |
| Control-FREEC | 5 | 9 | 9 | 10 |
| Control-FREEC | 10 | 9 | 10 | 10 |
| MetaCNV | 1 | 100 | 100 | 100 |
| MetaCNV | 2 | 100 | 100 | 100 |
| MetaCNV | 5 | 100 | 100 | 100 |
| MetaCNV | 10 | 100 | 100 | 100 |
| ReadDepth | 1 | 99 | 99 | 99 |
| ReadDepth | 2 | 99 | 99 | 99 |
| ReadDepth | 5 | 100 | 100 | 99 |
| ReadDepth | 10 | 99 | 99 | 99 |
| SVDetect | 1 | 94 | 94 | 94 |
| SVDetect | 2 | 94 | 94 | 94 |
| SVDetect | 5 | 94 | 94 | 94 |
| SVDetect | 10 | 94 | 94 | 94 |

## Callers’ prediction results and evaluations


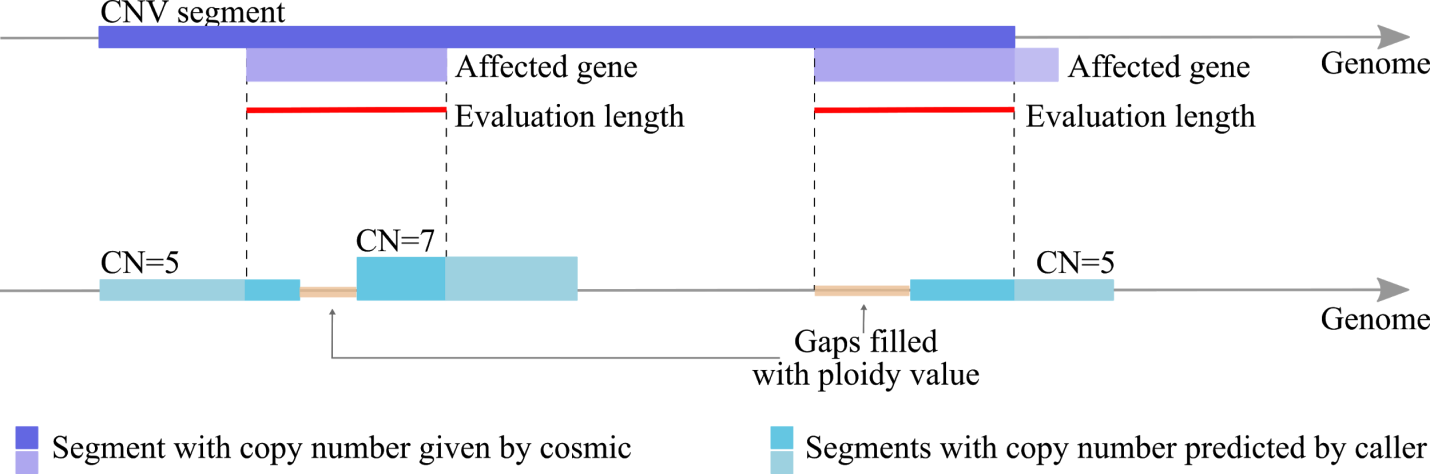


**Figure S9. Evaluation length and gaps in predictions.** If a mutated segment given by COSMIC does not completely cover a gene, the evaluation of a caller’s prediction accurateness was only considered for the reduced length, i.e. the evaluation length. If a caller did not predict a copy number for the complete evaluation length, the ploidy value was set for the length of the gap. Different copy number predictions for one gene were averaged considering the segment length, i.e. the sequence length per copy number, covering the gene.


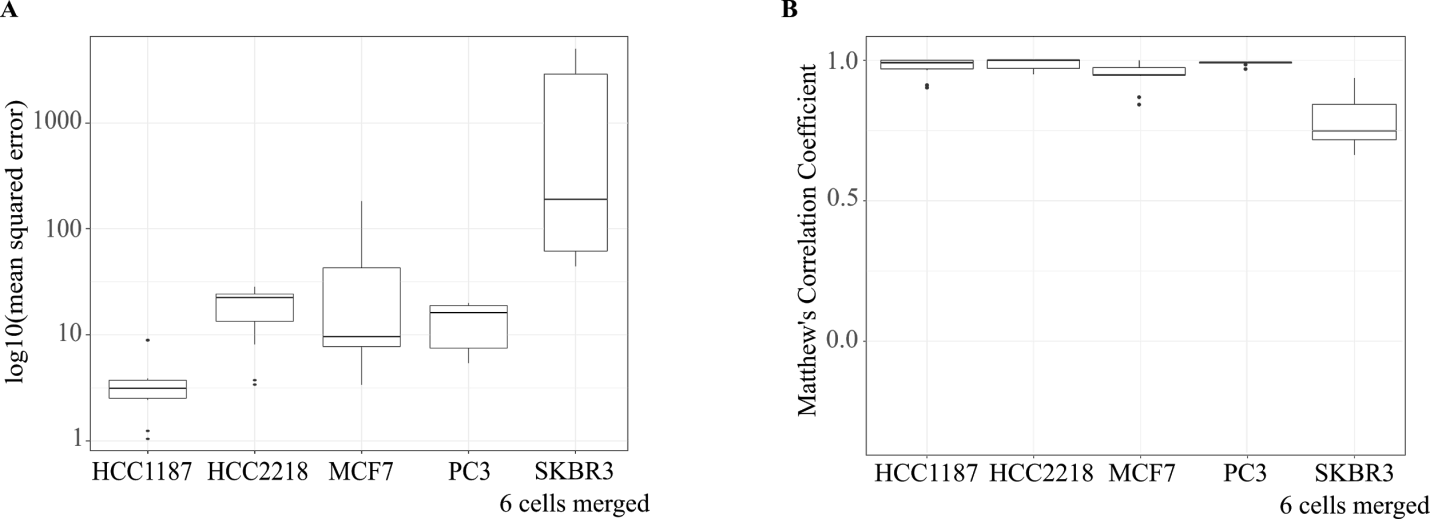


**Figure S10. The distribution of MSE and MCC values for the predicted copy numbers of all used methods (MetaCNV, SVDetect simNormal, ReadDepth, Control-FREEC, CopyCat, and CNVnator) per cell line.** The prediction results varied for high coverage data, but considerably more for low coverage data. For the HCC1187, HCC2218 with the highest read coverage among the evaluated cancer cell lines (104x & 93x) the callers mainly agreed in their predictions.
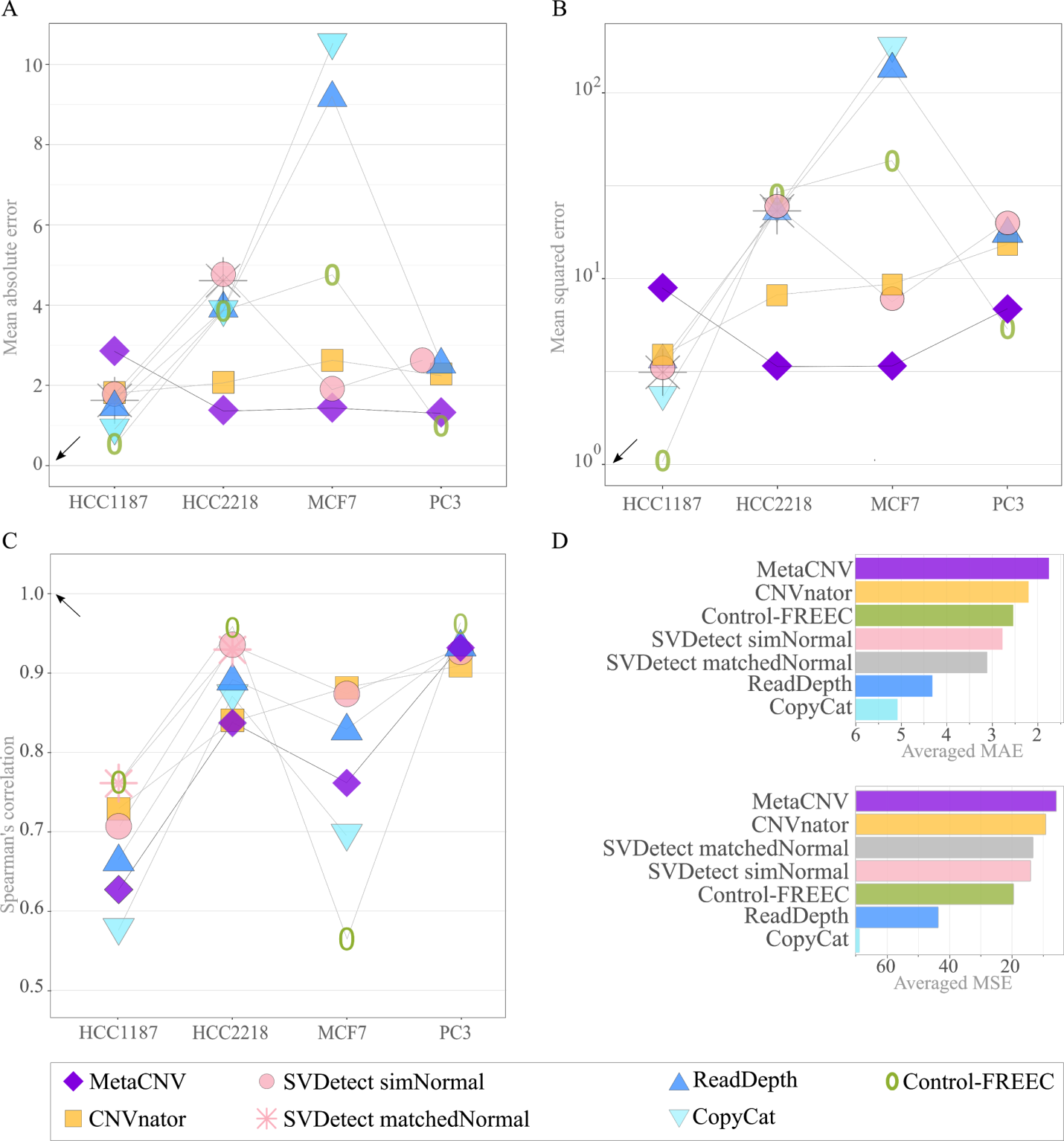


**Figure S11.** Mean absolute error (**A**), mean squared error (**B**), and Spearman’s correlation (**C**) for high coverage data. **D** presents the overall results for A and B averaged across all benchmarked cell lines.


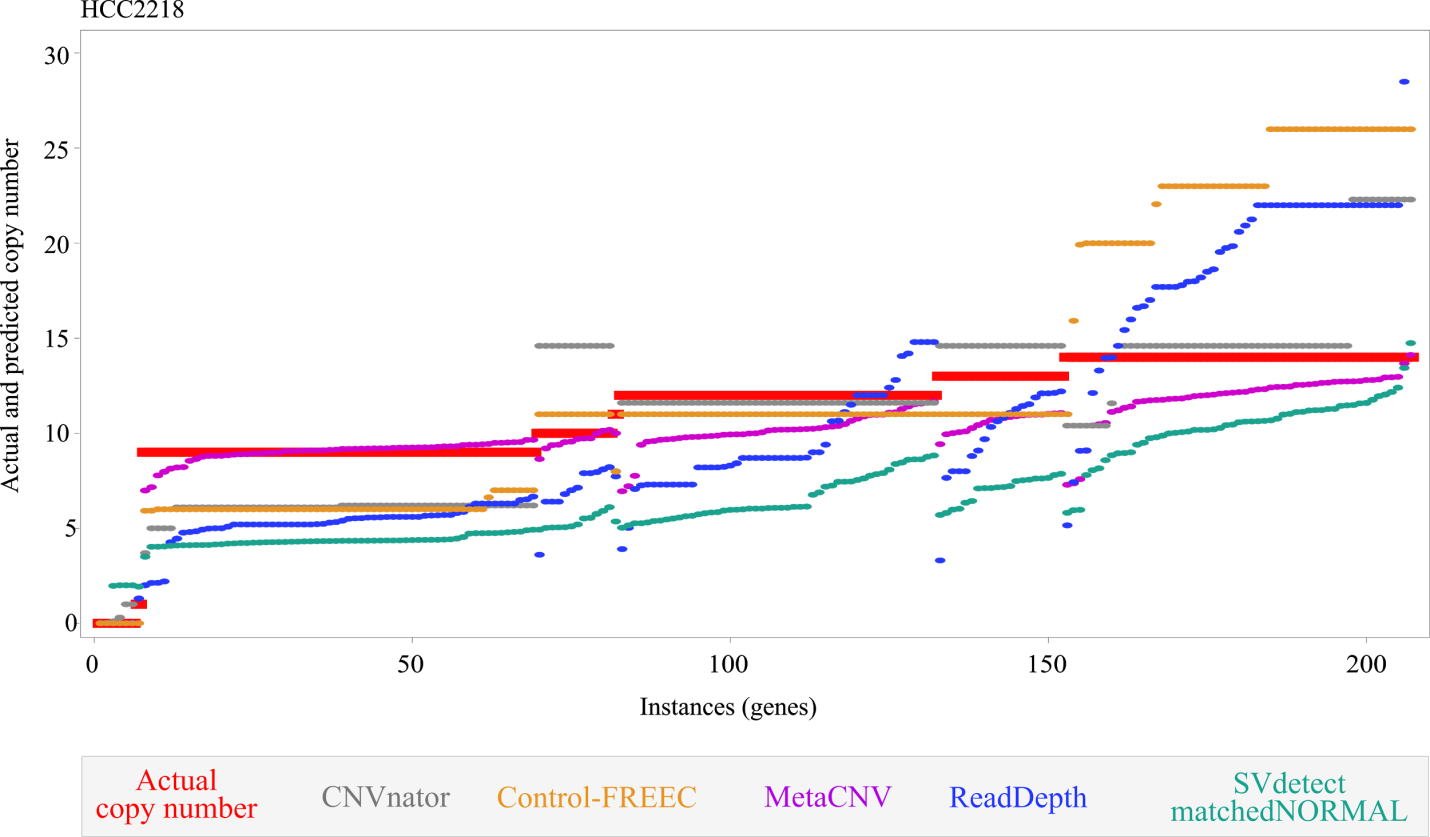


**Figure S12**. **Prediction results visualised for HCC2218.** For each caller, the predicted copy numbers were sorted independently by true copy numbers and within each true copy number by predicted copy number. It can be seen that MetaCNV predicts values close to the true copy numbers, i.e. low variance of residuals. For example, MetaCNV predicts a range of *CN_Met_*_a_={7 ..14} for true copy numbers *CN_tru_*_e_=14 and a *CN_Meta_*=14 only for true copy numbers *CN_true_*=14; whereas Control-FREEC predicts different copy numbers for genes with a *CN_true_*=14 (11 < *CN_SVDetect_* < 26) and a *CN_SVDetect_*=11 was predicted for genes with *CN_true_*={10, 12, 13}.

## Low coverage data


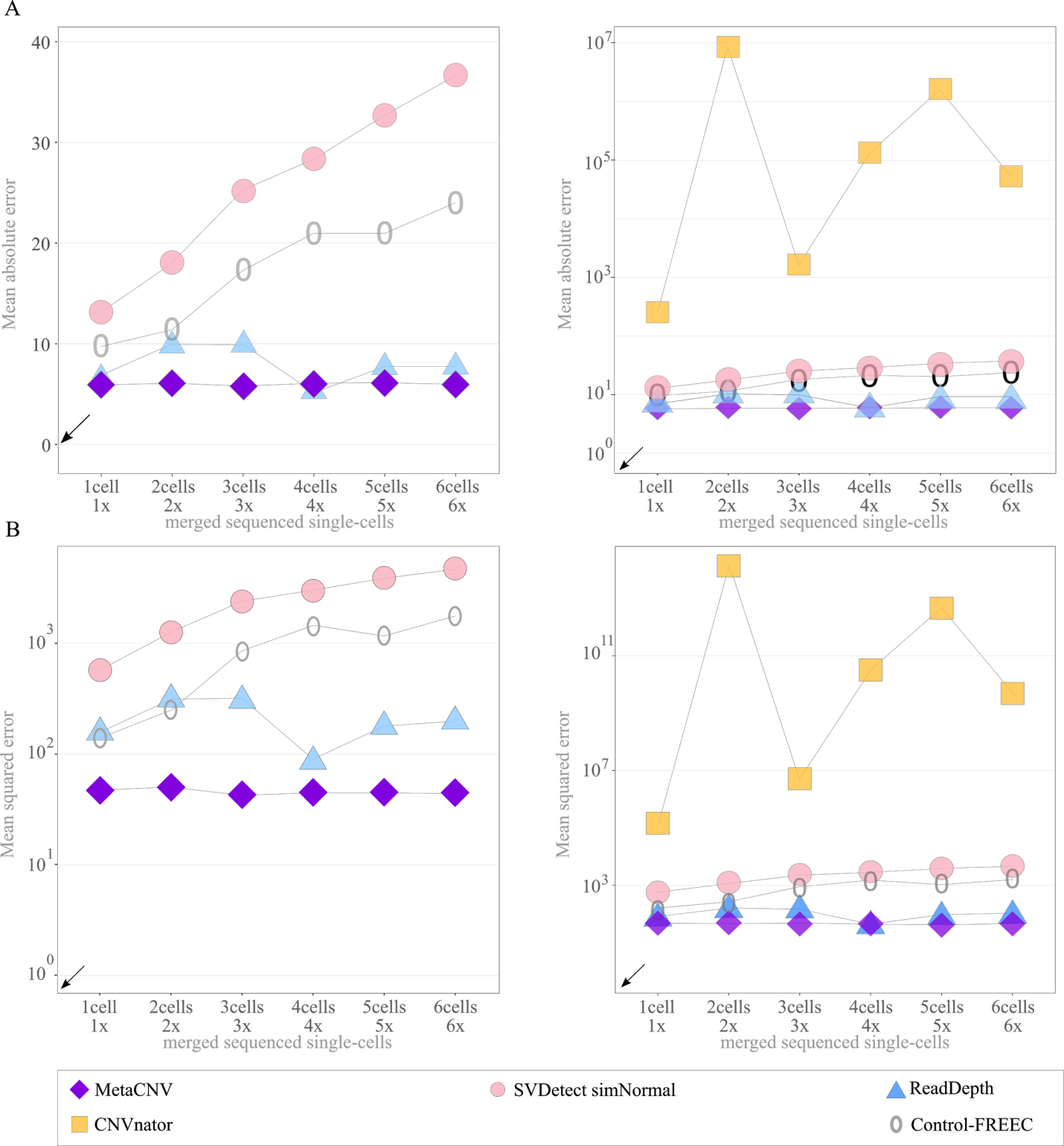


**Figure S13.** **Additional error benchmarks for low coverage data.** Mean absolute error (A), mean squared error (B) for single sequenced cell line SKBR3. Both error measures are shown without (left) and with CNVnator (right)


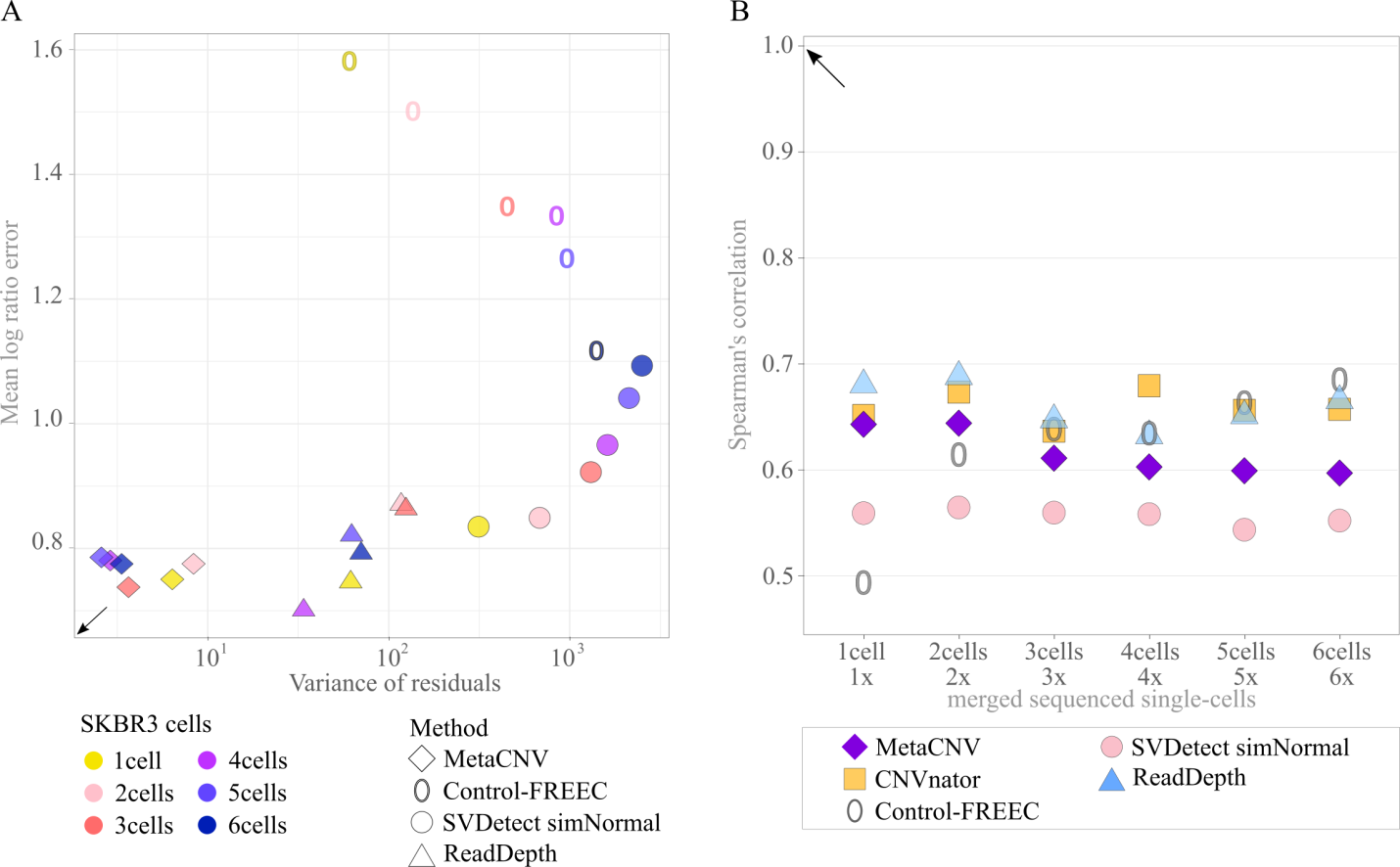


**Figure S14. Additional benchmarks for low coverage data. A** Mean log ratio error (y-axis) versus variance of residuals (x-axis) for low coverage data. **B** Spearman's correlation for low coverage data.


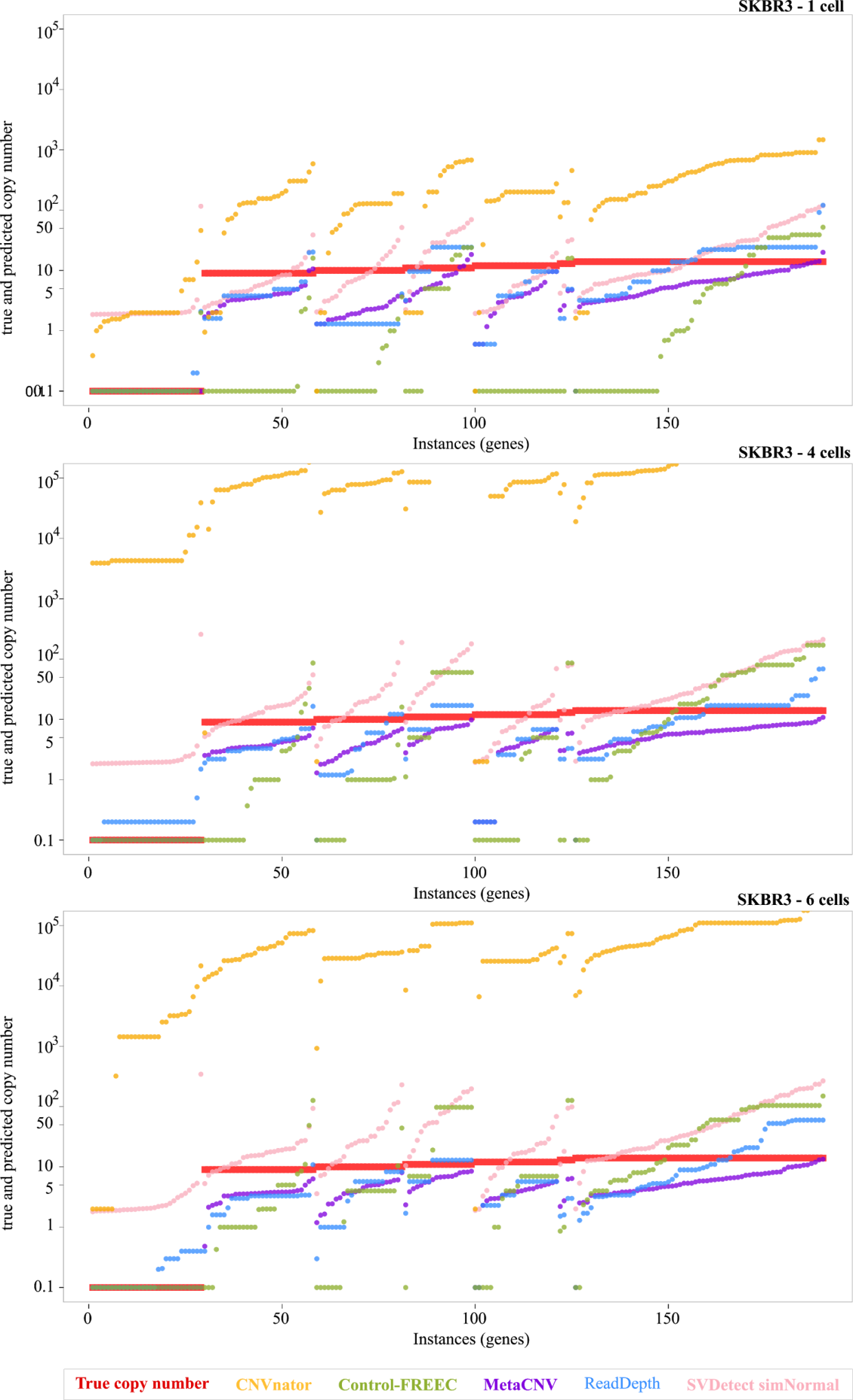


**Figure S15. Prediction results visualised for cell line SKBR3.** With increasing coverage (1x to 6x), less genes were predicted as deleted, simultaneously, more predicted copy numbers exceeded the true copy numbers. For each caller, the predicted copy numbers were sorted independently by true copy numbers and within each true copy number by predicted copy number. Copy numbers < 0.1 were replaced by 0.1 to limit the log(*CN*) value to -1.

## Simulated mutated genomes


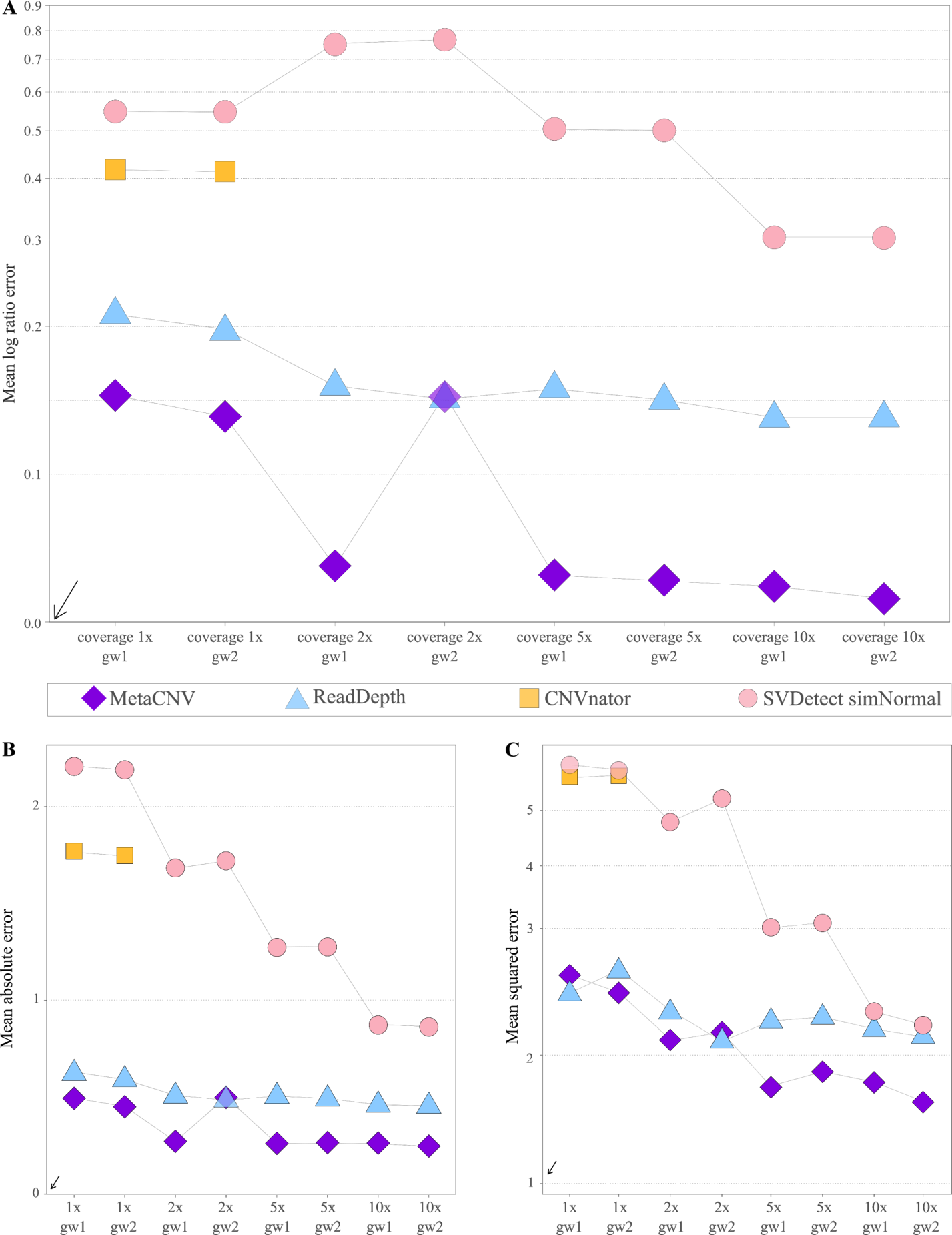


**Figure S16.** Benchmarks for the simulated mutated genomes (gw1 and gw2) for which genome-wide predicted copy numbers by MetaCNV, SVDetect, ReadDepth, and CNVnator were available. CNVnator, however, predicted genome-wide copy numbers only for the simulated 1x genomes (Table S4). **A** Mean log ratio MLRE. **B** Mean absolute error MAE. **C** Mean squared error MSE.


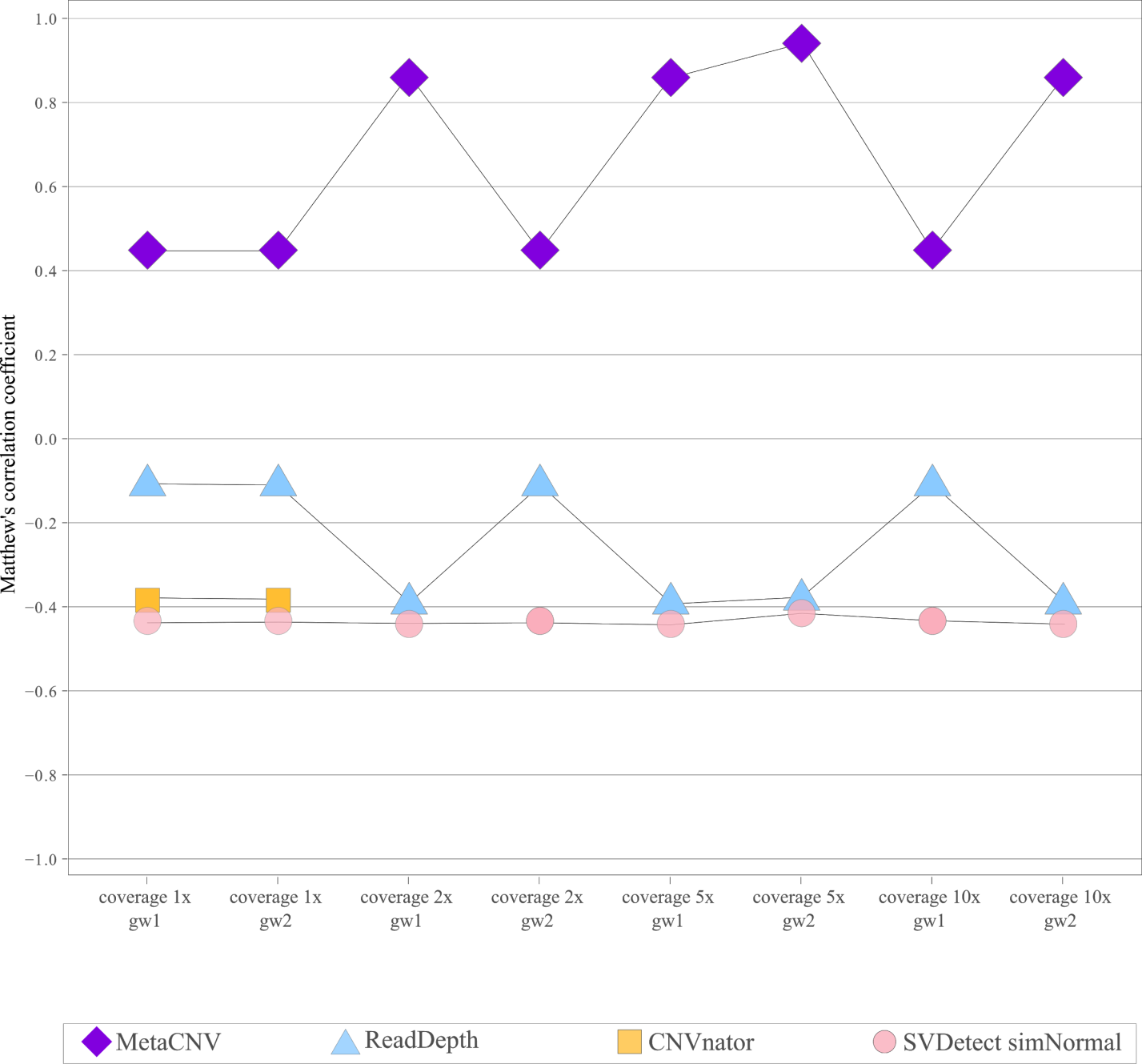


**Figure S17.** MCC for the simulated mutated genomes (gw1 and gw2) for which genome-wide predicted copy numbers by MetaCNV, SVDetect, ReadDepth, and CNVnator were available. CNVnator, however, predicted genome-wide copy numbers only for the simulated 1x genomes (Table S4).

**Table S5.** Variance of residuals for the simulated genomes used for genome-wide prediction (gw1 and gw2). CNVnator predicted genome-wide copy numbers only for the 1x genomes (Table S4)

| Method | 1x  gw1 | 1x  gw2 | 2x  gw1 | 2x  gw2 | 5x  gw1 | 5x  gw2 | 10x  gw1 | 10x  gw2 |
| --- | --- | --- | --- | --- | --- | --- | --- | --- |
| CNVnator | 6.0 | 6.1 |  |  |  |  |  |  |
| MetaCNV | 6.6 | 3.1 | 6.7 | 1.6 | 0.2 | 0.2 | 0.2 | 0.2 |
| ReadDepth | 56.2 | 61.2 | 20.8 | 11.6 | 14.6 | 11.7 | 4.0 | 4.8 |
| SVDetect | 21.4 | 18.5 | 0.3 | 0.3 | 1.3 | 1.4 | 2.1 | 2.0 |

**Table S6.** Spearman’s correlation for the simulated mutated genomes used for genome-wide prediction of copy numbers. CNVnator predicted genome-wide copy numbers only for the 1x genomes (Table S4)

| Method | 1x  gw1 | 1x  gw2 | 2x  gw1 | 2x  gw2 | 5x  gw1 | 5x  gw2 | 10x  gw1 | 10x  gw2 |
| --- | --- | --- | --- | --- | --- | --- | --- | --- |
| CNVnator | 0.36 | 0.36 |  |  |  |  |  |  |
| MetaCNV | 0.30 | 0.31 | 0.42 | 0.43 | 0.54 | 0.45 | 0.45 | 0.44 |
| ReadDepth | 0.27 | 0.26 | 0.41 | 0.43 | 0.42 | 0.44 | 0.55 | 0.49 |
| SVDetect | 0.39 | 0.39 | 0.38 | 0.05 | 0.39 | 0.40 | 0.39 | 0.38 |


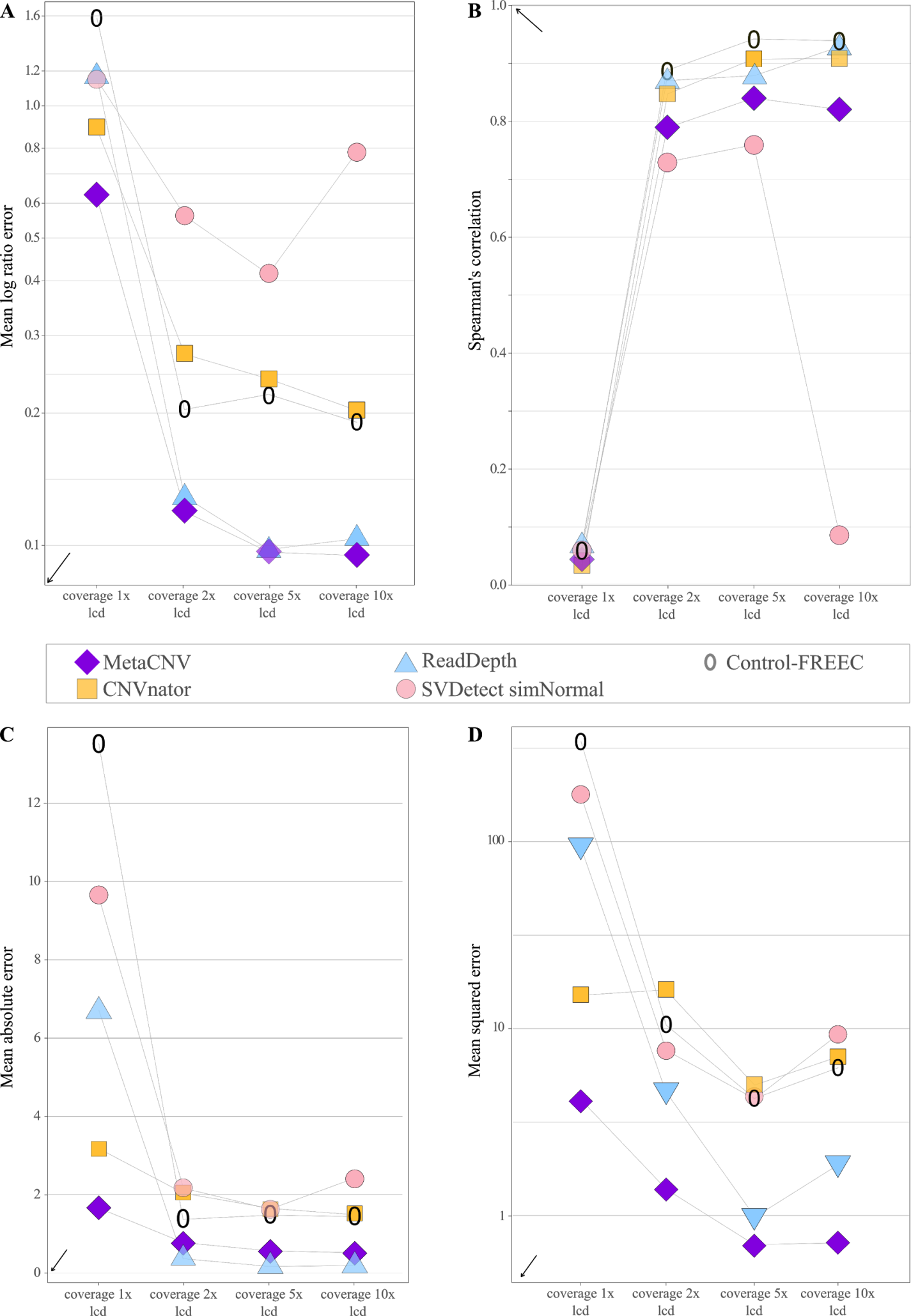


**Figure S18.** Benchmarks for the simulated geomes reduced to genes for which a prediction of the tested callers MetaCNV, SVDetect, ReadDepth, CNVnator, and Control-FREEC was available. **A** Mean log ratio error MLRE. **B** Spearman’s correlation coefficient. **C** Mean absolute error. **D** Mean squared error

**Table S7.** Variance of residuals for the simulated genomes reduced to genes for which a prediction of the callers MetaCNV, SVDetect, ReadDepth, CNVnator, and Control-FREEC was available

| Method | Coverage 1x  lcd | Coverage 2x  lcd | Coverage 5x  lcd | Coverage 10x  lcd |
| --- | --- | --- | --- | --- |
| CNVnator | 11.7 | 14.3 | 1.3 | 7.6 |
| Control-FREEC | 237.1 | 11.9 | 1.2 | 7.6 |
| MetaCNV | 4.1 | 0.7 | 0.2 | 0.7 |
| ReadDepth | 82.4 | 7.4 | 1.2 | 6.1 |
| SVDetect | 93.1 | 0.1 | 0.2 | 0.0 |


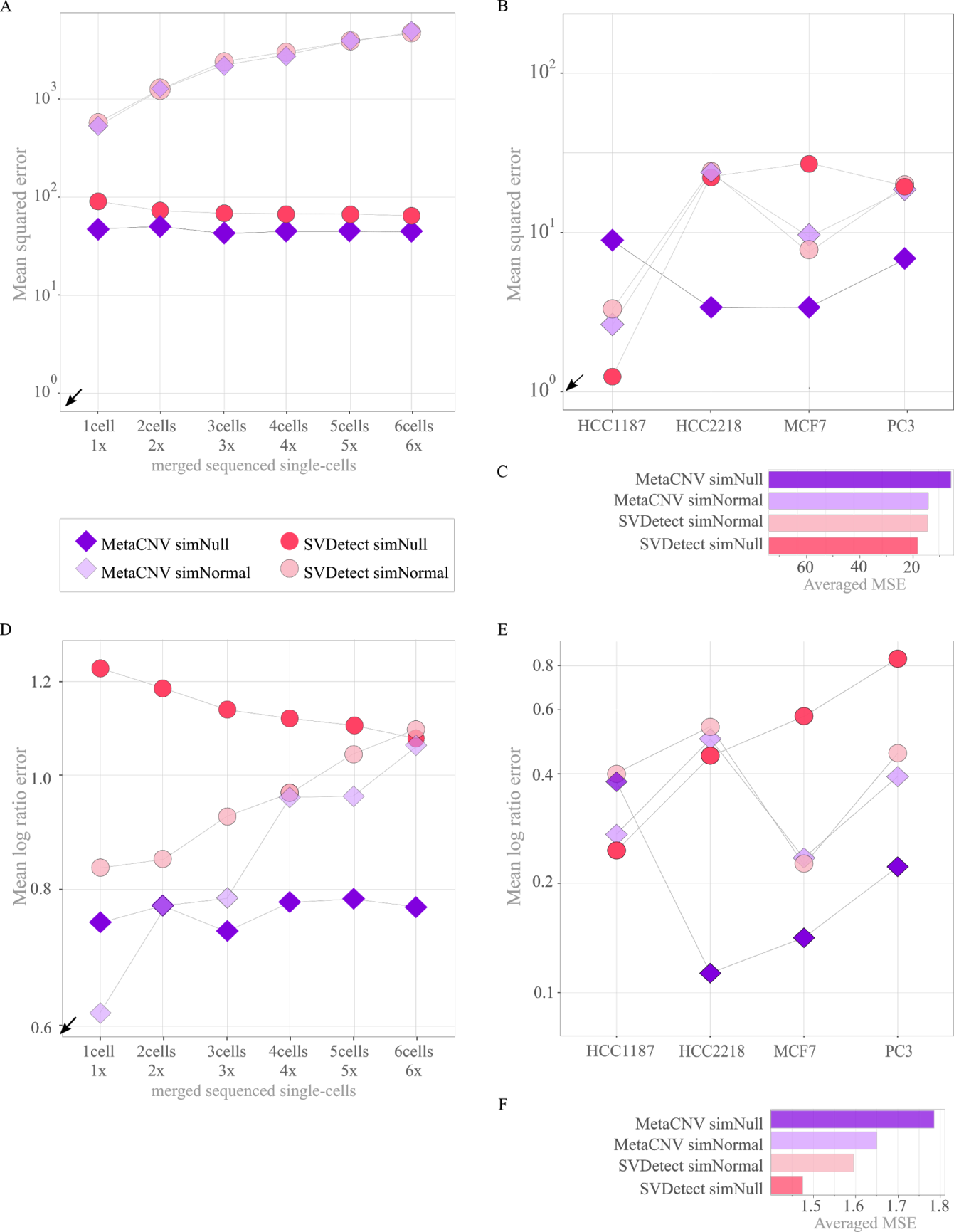


**Figure S19. Comparison of MetaCNV run with simNull and with simNormal** for both low (A) and high coverage data (B). The Input of MetaCNV simNull (default) is ReadDepth and SVDetect using simNull as matched sample; the input of MetaCNV simNormal is also ReadDepth but SVDetect using simNormal as matched sample.


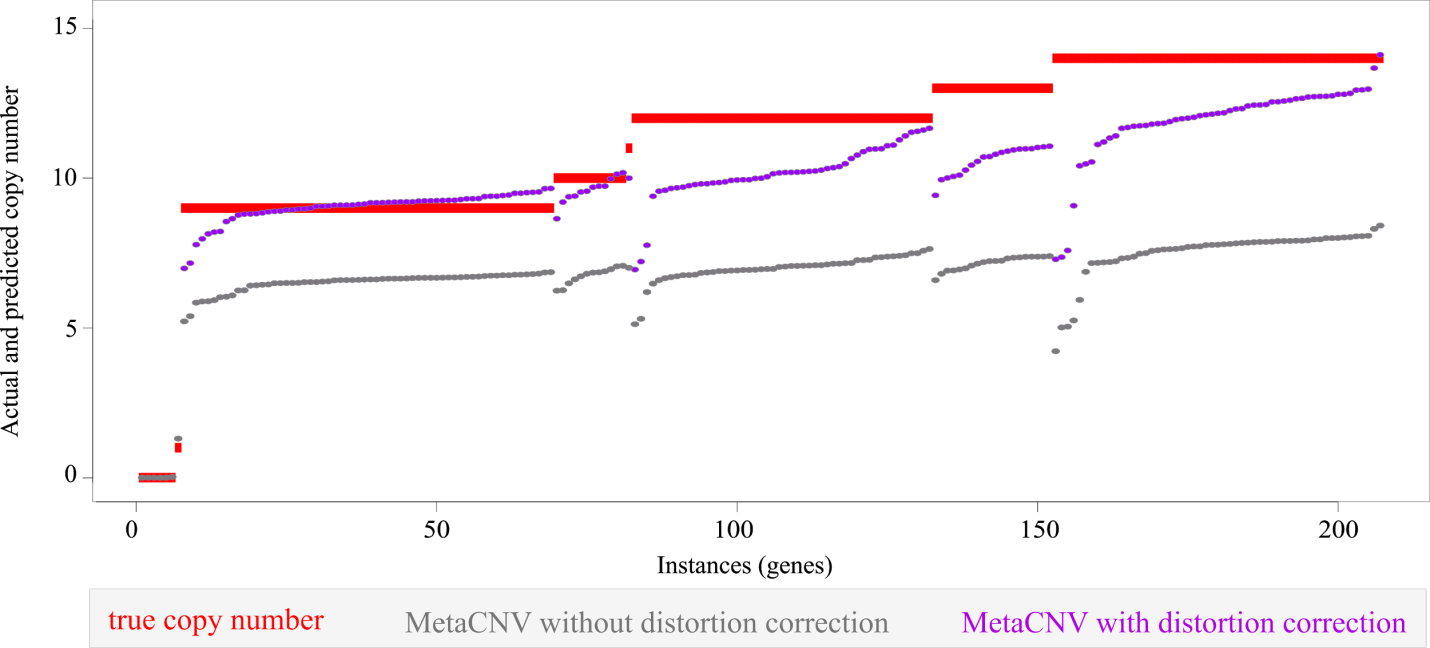


**Figure S20.** **Prediction result of MetaCVN with and without distortion correction (equalizer factor q) visualised for HCC2218**. For each caller, the predicted copy numbers were sorted independently by true copy number and within each true copy number by predicted copy number.

## Bias and Variances

**Table S8. Bias (systematic error) per caller for single sequenced cell line SKBR3**

| Method | 1 cell | 2 cells | 3 cells | 4 cells | 5 cells | 6 cells |
| --- | --- | --- | --- | --- | --- | --- |
| CNVnator | -2.51 * 10^2^ | -8.48 *10^6^ | -1.65 * 10^3^ | -1.34 * 10^5^ | -1.62 * 10^6^ | -5.28 * 10^4^ |
| Control-FREEC | 4.83 | 1.78 | -6.74 | -10.84 | -11.69 | -16.11 |
| MetaCNV | 5.55 | 5.39 | 5.65 | 5.8 | 5.88 | 5.71 |
| ReadDepth | 0.55 | -1.59 | -1.81 | 2.21 | 0.86 | 0.71 |
| SVDetect simNormal | -7.68 | -14.55 | -22.95 | -26.46 | -31.07 | -35.58 |

**Table S9. Variance (random error) per caller for single sequenced cell line SKBR3**

| Method | 1 cell | 2 cells | 3 cells | 4 cells | 5 cells | 6 cells |
| --- | --- | --- | --- | --- | --- | --- |
| CNVnator | 85.15 * 10^3^ | 63.38 * 10^12^ | 2.48 * 10^6^ | 13.95 * 10^9^ | 1.91 * 10^12^ | 2.07 * 10^9^ |
| Control-FREEC | 124.69 | 273.64 | 853.82 | 1373.02 | 1108.8 | 1579.19 |
| MetaCNV | 14.09 | 19.59 | 10.11 | 6.63 | 6.4 | 7.49 |
| ReadDepth | 182.56 | 349.03 | 338.83 | 97.41 | 204.87 | 225.8 |
| SVDetect simNormal | 565.51 | 1121.12 | 1976.47 | 2416.75 | 3091.69 | 3632.89 |

**Table S10. Variance of the residuals per caller for single sequenced cell line SKBR3**

| Method | 1 cell | 2 cells | 3 cells | 4 cells | 5 cells | 6 cells |
| --- | --- | --- | --- | --- | --- | --- |
| CNVnator | 3.49 * 10^4^ | 2.66 * 10^13^ | 1.02 * 10^6^ | 7.86 * 10^9^ | 7.05 * 10^11^ | 7.09 * 10^8^ |
| Control-FREEC | 60.73 | 136.41 | 452.55 | 847.18 | 966.47 | 1407.13 |
| MetaCNV | 6.37 | 8.35 | 3.65 | 2.89 | 2.58 | 3.34 |
| ReadDepth | 61.63 | 117.07 | 124.58 | 33.88 | 62.36 | 70.33 |
| SVDetect simNormal | 313.51 | 682.23 | 1308.59 | 1616.51 | 2125.14 | 2508.49 |

**Table S11. Bias (systematic error) per caller for high coverage data**

| Method | HCC1187 | HCC2218 | MCF7 | PC3 |
| --- | --- | --- | --- | --- |
| CNVnator | 1.60 | 0.19 | -0.26 | 2.28 |
| CopyCat | 0.81 | -0.21 | -8.12 | NA |
| Control-FREEC | -0.13 | -1.19 | -1.42 | 0.97 |
| MetaCNV | -2.82 | 1.27 | 1.35 | 1.33 |
| ReadDepth | 1.47 | 0.82 | -7.55 | 2.57 |
| SVDetect matched NORMAL | 1.56 | 4.51 |  |  |
| SVDetect simNORMAL | 1.43 | 4.64 | 0.91 | 2.63 |

**Table S12. Variance (random error) per caller for high coverage data**

| Method | HCC1187 | HCC2218 | MCF7 | PC3 |
| --- | --- | --- | --- | --- |
| CNVnator | 0.58 | 22.01 | 34.53 | 7.17 |
| CopyCat | 2.93 | 46.09 | 180.33 | NA |
| Control-FREEC | 3.78 | 51.80 | 64.77 | 16.09 |
| MetaCNV | 6.05 | 5.36 | 14.47 | 13.25 |
| ReadDepth | 1.52 | 41.10 | 136.36 | 6.53 |
| SVDetect matchedNORMAL | 1.22 | 7.36 |  |  |
| SVDetect simNORMAL | 0.41 | 6.78 | 28.73 | 4.84 |

**Table S13. Variance of the residuals per caller for high coverage data**

| Method | HCC1187 | HCC2218 | MCF7 | PC3 |
| --- | --- | --- | --- | --- |
| CNVnator | 0.12 | 2.20 | 3.48 | 0.44 |
| CopyCat | 0.70 | 7.99 | 57.42 | NA |
| Control-FREEC | 0.68 | 1.58 | 18.47 | 2.24 |
| MetaCNV | 0.29 | 0.57 | 0.80 | 0.50 |
| ReadDepth | 0.36 | 6.27 | 20.95 | 0.78 |
| SVDetect matchedNORMAL | 0.20 | 0.97 |  |  |
| SVDetect simNORMAL | 0.14 | 0.75 | 3.25 | 0.92 |

**Table S14.** Benchmark results for MetaCNV on cancer cell line PC3 aligned with Bowtie2 or BWA

| MetaCNV | MSE | MAE | MCC | MLRE |
| --- | --- | --- | --- | --- |
| PC3 Bowtie2 aligned 76x | 6.87 | 1.70 | 0.98 | 0.22 |
| PC3 BWA aligned 77x | 6.87 | 1.67 | 0.99 | 0.21 |

## Pre-processing and application of calling methods

### Alignment & pre-processing of the alignments

Alignment of raw reads was performed using Bowtie2 v2.2.9 [36], removal of duplicates using Picard v1.119 [37], converting, sorting and indexing using Samtools v1.2 [38].

### Copy number calling with ReadDepth

Bam to bed conversion followed by pre-processing was done according to the recommendations of ReadDepth v0.9.8.4. ReadDepth was run with default parameters (false discovery rate, fdr 0.01) on the cell lines HCC1187, HCC2218, MCF7, PC3, and the single cell sequenced SKBR3 (example file with parameters and script to run readDepth available in the Bitbucket repository in /Examples). We tested increased FDRs to decrease the bin size but found that the default value of 0.01 is sufficient.

ReadDepth was run with the option ‘removeGaps=False’ to test if there are regions that are actually deleted but due to smoothing were set as non-deleted; this was not confirmed. Running with removeGaps=TRUE achieves better results for the tested cancer cell lines. Although there are rare large gaps in genome that could not be removed by ReadDepth, these are non-sequenced regions (no coverage in non-genetic regions with visual check of the alignment).

Annotations (entrypoints, gcWinds, and mapability) for the respective read length of each cancer cell line were downloaded from <https://github.com/chrisamiller/readDepth> or created with the provided bash script.

**Table S15.** Top 20 rows of ReadDepth output for SKBR3 single cell 1 (1x coverage). Bold column names were used as MetaCNV input for copy number calculation.

| Chr | Segment start coordinate | Segment end coordinate | Bins | **Copy number** |
| --- | --- | --- | --- | --- |
| chr1 | 1 | 49831500 | 1995 | **0.30** |
| chr1 | 49831501 | 54085700 | 178 | **0.59** |
| chr1 | 54085701 | 54659300 | 24 | **7.90** |
| chr1 | 54659301 | 55089500 | 18 | **0.36** |
| chr1 | 55089501 | 55543600 | 19 | **0.03** |
| chr1 | 55543601 | 62594100 | 295 | **0.75** |
| chr1 | 62594101 | 62904800 | 13 | **0.01** |
| chr1 | 62904801 | 94046500 | 1300 | **0.85** |
| chr1 | 94046501 | 94548400 | 21 | **0.04** |
| chr1 | 94548401 | 110633100 | 648 | **0.85** |
| chr1 | 110633101 | 111182800 | 23 | **0.04** |
| chr1 | 111182801 | 112043200 | 36 | **2.00** |
| chr1 | 112043201 | 113811800 | 74 | **0.16** |
| chr1 | 113811801 | 114552700 | 31 | **2.40** |
| chr1 | 114552701 | 114791700 | 10 | **0.03** |
| chr1 | 114791701 | 121352250 | 254 | **0.82** |
| chr1 | 142551550 | 143161000 | 9 | **0.00** |
| chr1 | 143161001 | 153461900 | 260 | **0.59** |
| chr1 | 153461901 | 225998400 | 2998 | **0.96** |
| chr1 | 225998401 | 248956422 | 943 | **0.57** |

### Copy number calling with SVDetect

For SVDetect v1.3 different bin sizes (400, 500, 1000, and 10000; each with an overlap of 10 or 250, and without overlap) were tested. Using a window of 400 bp with no overlap achieved the best results. The same chromosome lengths (entrypoints) as for ReadDepth were used. An example file with parameters and script to run SVDetect is available in the Bitbucket repository in /Examples.

#### Gaps in SVDetect

As in the ReadDepth result, some gaps might be caused by non-sequenced regions. Otherwise, SVDetect outputs the most gaps with a multiple length of the set bin size (400 bp). There is no obvious reason why SVDetect introduced these gaps; the coverage is sufficient. Rare gaps larger than 800 bp are regions which were probably not sequenced (no coverage in non-genetic regions with a visual check of the alignment).

**Table S16 .** Top 20 rows of SVDetect output for SKBR3 single cell 1 (1x coverage). Bold column names were used as MetaCNV input for copy number calculation.

| Chr | Segment start coordinate | Segment end coordinate | Average depth-of-coverage from the sample paired-end data | Average depth-of-coverage from the reference paired-end data | **Log-ratio of 4. and 5. values** |
| --- | --- | --- | --- | --- | --- |
| 1 | 12801 | 13200 | 1 | 0 | **0.69** |
| 1 | 13201 | 13600 | 2 | 0 | **1.09** |
| 1 | 13601 | 14000 | 1 | 0 | **0.69** |
| 1 | 14801 | 15200 | 3 | 0 | **1.38** |
| 1 | 15201 | 15600 | 3 | 0 | **1.38** |
| 1 | 16001 | 16400 | 32 | 0 | **3.49** |
| 1 | 16401 | 16800 | 43 | 0 | **3.78** |
| 1 | 16801 | 17200 | 84 | 0 | **4.44** |
| 1 | 17201 | 17600 | 116 | 0 | **4.76** |
| 1 | 17601 | 18000 | 18 | 0 | **2.94** |
| 1 | 18001 | 18400 | 8 | 0 | **2.19** |
| 1 | 18401 | 18800 | 10 | 0 | **2.39** |
| 1 | 18801 | 19200 | 3 | 0 | **1.38** |
| 1 | 19201 | 19600 | 86 | 0 | **4.46** |
| 1 | 19601 | 20000 | 91 | 0 | **4.52** |
| 1 | 20001 | 20400 | 120 | 0 | **4.79** |
| 1 | 20401 | 20800 | 355 | 0 | **5.87** |

### Copy number calling with CNVnator

CNVnator v0.3.2 was run in five steps (creating root file, making histogram, calculating statistics, partitioning, and identifying CNVs) according to the CNVnator recommendations. Bin size was set to 400 as this bin size was also chosen for SVDetect. An example script to run CNVnator is available in the Bitbucket repository in /Examples.

**Table S17.** First lines of CNVnator output for SKBR3 single cell 1 (1x coverage). Bold column names were used as MetaCNV input for copy number calculation. The content of column “Copy number (normalised to 1)” is not reliable because it contradicts with the column “Copy number type”.

| **Copy number type** | Chr:  segment start- segment end | Segment length | Copy number (normalised to 1) | p-val1 | p-val2 | p-val3 | p-val3 | q0 |
| --- | --- | --- | --- | --- | --- | --- | --- | --- |
| **deletion** | 1:1-13200 | 13200 | 0.08 | 1.56958E-10 | 723515 | 1.20857E-08 | 3263890 | 1 |
| **deletion** | 1:208001-259200 | 51200 | 0.04 | 3.11274E-12 | 2859680000 | 680.958 | 1.0375E-84 | 1 |
| **deletion** | 1:293201-350000 | 56800 | 0.00 | 360.246 | 4.98447E-100 | 434.693 | 1.96395E-95 | 1 |
| **deletion** | 1:374001-382400 | 8400 | 0.00 | 60036.6 | 2.36454E-07 | 103694 | 0.00931659 | 1 |
| **deletion** | 1:395601-410000 | 14400 | 0.00 | 21908.4 | 7.67309E-19 | 31503.6 | 3.0233E-14 | 1 |
| **deletion** | 1:530401-586400 | 56000 | 0.07 | 2.84594E-12 | 2871000000 | 463.045 | 6.68293E-94 | 1 |
| **deletion** | 1:602401-611600 | 9200 | 0.00 | 51300.6 | 6.9488E-09 | 85120.7 | 0.000273792 | 1 |
| **deletion** | 1:715201-725200 | 10000 | 0.05 | 4.14369E-10 | 34508.9 | 71020.6 | 8.04606E-06 | 1 |
| **deletion** | 1:752801-758800 | 6000 | 0.00 | 103694 | 0.00931659 | 219126 | 367.086 | 1 |
| **deletion** | 1:774801-788400 | 13600 | 0.14 | 0.000229786 | 1088670000 | 0.000597266 | 1291860000 | 1 |
| **deletion** | 1:937601-946800 | 9200 | 0.00 | 51300.6 | 6.9488E-09 | 85120.7 | 0.000273792 | 1 |
| **deletion** | 1:1157201-1175200 | 18000 | 0.13 | 0.0075837 | 2870940000 | 0.349646 | 2870950000 | 1 |
| **deletion** | 1:1239601-1440000 | 200400 | 7.69 | 6.45365 | 3.47942E-32 | 6.38796 | 1.07529E-31 | 1 |
| **deletion** | 1:1532401-1541600 | 9200 | 0.10 | 0.0020657 | 322936000 | 85120.7 | 0.000273792 | 1 |
| **deletion** | 1:1614801-1624800 | 10000 | 0.11 | 8.01685E-06 | 1742780 | 0.0303349 | 10310300 | 1 |
| **duplication** | 1:1726401-1786400 | 60000 | 68.84 | 0.0369587 | 0.00161278 | 0.00493937 | 0.00498419 | 1 |

### Copy number calling with Control-FREEC

For Control-FREEC v8.0, the same bin size of 400 was set. The same entry points (genome length) were used as for ReadDepth and SVDetect.

### Copy number calling with CopyCat

CopyCat v1.6.11 was run as single sample approach. The parameters were set similar to the ones for ReadDepth. CopyCat could not be run on cancer cell PC3 which was probably caused by a format error in the alignment which we could not resolve.

### Simulated normal data


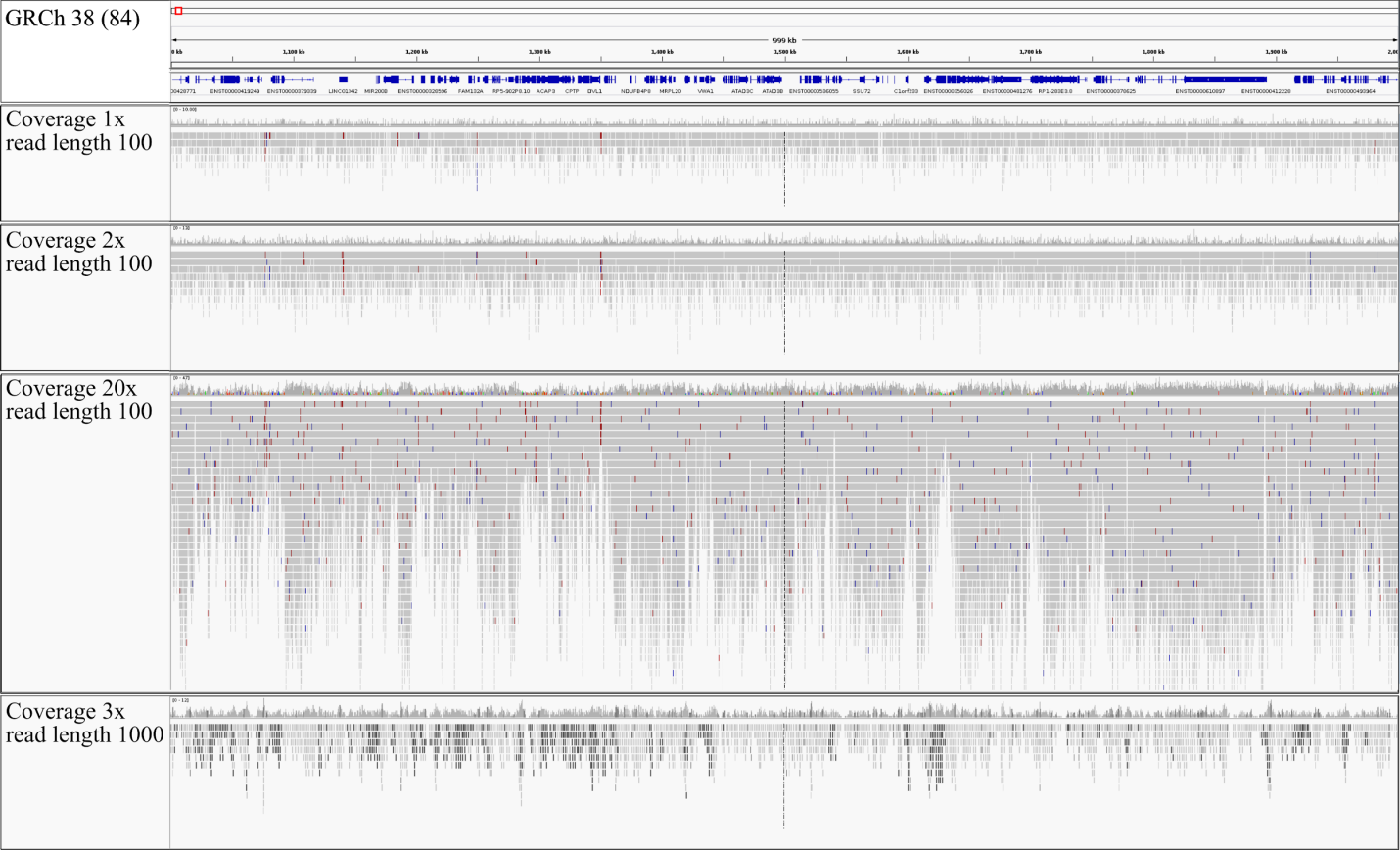
**Figure S21. Aligned simulated reads of different coverages (1x, 2x, 3x, 20x) and different read lengths (199 bp, 1000 bp) using Pirs 2.0.** Although each simulation was done with constant coverage, a high variance in coverage can be observed, which is independent from the read length. The simulated alignment with 20x and read length of 100 bp was used as a simNormal sample for callers that require a matched sample for copy number calling (e.g SVDetect). The normal alignment (simNormal) was simulated with Pirs [23] and default parameters plus a read length of 100 bp and a coverage of 20x. The simulated null alignment (simNull) is an alignment file in *.bam format with zero coverage.

## MetaCNV’s v1.4 rules in detail

For each bin, the normalised copy numbers of the input callers were inspected and these rules applied.

**Table S18. MetaCNV’s rules in detail.**

| **Rule** | **Prediction** |
| --- | --- |
| If ReadDepth and SVDetect predicted nothing; | *CN_metaCNV_* = 0.0 |
| If ReadDepth predicted a value, but SVDetect did not | *CN_metaCNV_* = *CN_RD_* |
| If both, ReadDepth and SVDetect predicted a value |  |
| - If ReadDepth predicted a value < *T2* and SVDetect predicted a value > *T2* (conflict) | *CN_metaCNV_* = *CN_RD_* or *CN_SV_* depending on CNVnator |
| - If ReadDepth predicted a value > *T2* and SVDetect predicted a value < *T2* (conflict) | *CN_metaCNV_* = *CN_RD_* or *CN_SV_* depending on CNVnator |
| - If ReadDepth predicted a value < *T2* and SVDetect predicted a value < *T2* (agreement) | *CN_metaCNV_* = *CN_RD_* |
| - If ReadDepth predicted a value > *T1* & SVDetect predicted a value > *T2* (agreement) | *CN_metaCNV_* = *CN_SVDetect_* |
| If ReadDepth predicted nothing but SVDetect did |  |
| - If *CN_SVDetect_* < *T2* | *CN_metaCNV_* = 0.0 |
| - If *CN_SVDetect_* > *T2* | *CN_metaCNV_* = *CN_SVDetect_* |

## MetaCNV’s output

**Table S19. First lines of MetaCNV output for SKBR3 single cell 1 (1x coverage)**

| Chr | Segment start | Segment end | Segment. length | MetaCNV cn | MetaCNV error score | MetaCNV  comments array | RD cn array | SVDetect cn array | CNVnator type array |
| --- | --- | --- | --- | --- | --- | --- | --- | --- | --- |
| 1 | 1 | 1726400 | 1726399 | 0.3 | 20.2 | RD (SV is Null);  Conflict 2 (RD:DEL, SV:AMP);  Conflict 2 (RD:DEL, SV:AMP) judge: CNVnator:DEL | 0,30 | 0,00; 0,69; 1,09; 1,38; 3,49; 3,78; | not assigned; no data; deletion |
| 1 | 1726401 | 1726800 | 399 | 4.9 | 21.4 | Conflict 2 (RD:DEL, SV:AMP) judge: CNVnator:AMP | 0,30 | 4,92 | duplication |
| 1 | 1726801 | 1727200 | 399 | 3.8 | 12.4 | Conflict 2 (RD:DEL, SV:AMP) judge: CNVnator:AMP | 0,30 | 3,82 | duplication |
| 1 | 1727201 | 1728400 | 1199 | 0.3 | 1.2 | RD | 0,30 | 1,60; 0,69; 1,38 | not assigned |
| 1 | 1728401 | 1728800 | 399 | 3.8 | 12 | Conflict 2 (RD:DEL, SV:AMP) judge: CNVnator:AMP | 0,30 | 3,76 | duplication |
| 1 | 1728801 | 1729200 | 399 | 4.8 | 20 | Conflict 2 (RD:DEL, SV:AMP) judge: CNVnator:AMP | 0,30 | 4,77 | duplication |
| 1 | 1729201 | 1729600 | 399 | 5.3 | 25.4 | Conflict 2 (RD:DEL, SV:AMP) judge: CNVnator:AMP | 0,30 | 5,34 | duplication |

**Table S20. MetaCNV’s parameters & statistics for SKBR3 single cell 1 (1x coverage)**

| Gender |  | Female |
| --- | --- | --- |
| ReadDepth | infile | SKBR3_WGS_singleCell_3_readdepth.fdr0.01_segs.dat |
| ReadDepth | bias | 0 |
| ReadDepth | coverage | 97.46 % of GRCh38.84 |
|  |  |  |
| SVDetect | infile | SKBR3_WGS_singleCell_3_svdetect.w400.density |
| SVDetect | coverage | 11.27 % of GRCh38.84 |
|  |  |  |
| CNVnator | infile | SKBR3_WGS_singleCell_3_cnvnator.w400.csv |
| CNVnator | coverage | 54.28 % of GRCh38.84 |
|  |  |  |
| MetaCNV | outfile | SKBR3_female_cell3.dat |
| MetaCNV | coverage | 100.00 % of GRCh38.84 |
|  | Threshold for deletions | 1.8832 |
|  | Threshold for amplific. | 2.1168 |
